# Functional genomic analysis of epithelioid sarcoma reveals distinct proximal and distal subtype biology

**DOI:** 10.1002/ctm2.961

**Published:** 2022-07-15

**Authors:** Samuel V. Rasmussen, Jia xiang Jin, Lissett R. Bickford, Andrew D. Woods, Felix Sahm, Kenneth A. Crawford, Kiyo Nagamori, Hiroaki Goto, Keila E. Torres, Angelo Sidoni, Erin R. Rudzinski, Khin Thway, Robin L. Jones, Alessio Ciulli, Hollis Wright, Melvin Lathara, Ganapati Srinivasa, Kavya Kannan, Paul H. Huang, Thomas G. P. Grünewald, Noah E. Berlow, Charles Keller

**Affiliations:** ^1^ Children's Cancer Therapy Development Institute Beaverton Oregon USA; ^2^ Division of Translational Pediatric Sarcoma Research Hopp Children's Cancer Center (KiTZ), German Cancer Research Center (DKFZ) German Cancer Consortium (DKTK) Heidelberg Germany; ^3^ Department of Neuropathology, Institute of Pathology Heidelberg University Hospital Heidelberg Germany; ^4^ Clinical Cooperation Unit Neuropathology, German Consortium for Translational Cancer Research (DKTK), German Cancer Research Center (DKFZ) Heidelberg Germany; ^5^ Hopp Children's Cancer Center Heidelberg (KiTZ) Heidelberg Germany; ^6^ Division of Hematology/Oncology Kanagawa Children's Medical Center Yokohama Japan; ^7^ Sarcoma Surgical Oncology MD Anderson Cancer Center Houston Texas USA; ^8^ Section of Pathology, Department of Medicine and Surgery University of Perugia Perugia Italy; ^9^ Department of Pathology Seattle Children's Hospital Seattle Washington USA; ^10^ Sarcoma Unit, Royal Marsden Hospital Belmont UK; ^11^ Division of Molecular Pathology Institute of Cancer Research London UK; ^12^ Division of Clinical Studies Institute of Cancer Research London UK; ^13^ School of Life Sciences University of Dundee Dundee UK; ^14^ Omics Data Automation Beaverton Oregon USA

**Keywords:** distal, epithelioid sarcoma, functional genomics, proximal, SMARCB1

## Abstract

**Background:**

Metastatic epithelioid sarcoma (EPS) remains a largely unmet clinical need in children, adolescents and young adults despite the advent of EZH2 inhibitor tazemetostat.

**Methods:**

In order to realise consistently effective drug therapies, a functional genomics approach was used to identify key signalling pathway vulnerabilities in a spectrum of EPS patient samples. EPS biopsies/surgical resections and cell lines were studied by next‐generation DNA exome and RNA deep sequencing, then EPS cell cultures were tested against a panel of chemical probes to discover signalling pathway targets with the most significant contributions to EPS tumour cell maintenance.

**Results:**

Other biologically inspired functional interrogations of EPS cultures using gene knockdown or chemical probes demonstrated only limited to modest efficacy in vitro. However, our molecular studies uncovered distinguishing features (including retained dysfunctional *SMARCB1* expression and elevated *GLI3, FYN and CXCL12* expression) of distal, paediatric/young adult‐associated EPS versus proximal, adult‐associated EPS.

**Conclusions:**

Overall results highlight the complexity of the disease and a limited chemical space for therapeutic advancement. However, subtle differences between the two EPS subtypes highlight the biological disparities between younger and older EPS patients and emphasise the need to approach the two subtypes as molecularly and clinically distinct diseases.

## Graphical abstract

Metastatic Epithelioid Sarcoma has a largely unmet clinical need in pediatrics and young adults. To identify potential targets for inhibition we conducted next generation DNA exome and RNA deep sequencing and chemical screens in EPS patient samples and cell lines. We uncovered distinguishing features of the two subtypes of EPS, distal and proximal.

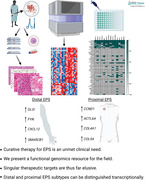

## 1 BACKGROUND

Epithelioid sarcoma (EPS) is a rare soft tissue sarcoma of children and young adults, which often presents as a seemingly benign growth, yet is very aggressive with metastatic spread occurring in up to 50% of cases with 1‐ and 5‐year survival rates of 46% and 0%, respectively.^1^, ^2^ EPS was first described by F.M. Enzinger in 1970 as a mesenchymal tumour with epithelioid‐like features.^3^ EPS is generally segmented into two distinct clinicopathological subgroups: the more prevalent distal (or classical) EPS which occurs in younger individuals (20–40 years of age), and proximal EPS which occurs predominantly in older populations (20–65 years of age).^4^, ^5^ Late relapse with metastases results in a significant unmet clinical need as surgery is not always possible and effective targeted therapies have yet to be discovered. Preclinical and translational research is needed to improve outcomes for patients with EPS, yet progress in EPS‐focused therapeutic development has been slow in part due to the paucity of EPS study models. Specifically, while several immortalised cell lines^6^ and patient samples exist globally, one centralised EPS biobank has not historically existed. The genomic and transcriptomic landscape are defined in only a limited number of biopsies,^7^ but even those data sets are not shared publicly. Furthermore, to our knowledge, few patient‐derived xenograft (PDX) mouse models exist for testing promising therapeutics,^8^, ^9^ highlighting multiple opportunities for this challenging disease. Recent clinical trials have identified tazemetostat, an *EZH2* inhibitor, as promising monotherapy treatment for proximal and distal EPS, with 9 of 62 epithelioid sarcoma patients (∼15%) demonstrating objective response to tazemetostat, although all responders were classified as partial responders. Median progression‐free survival and overall survival was 5.5 months and 19 months, respectively.^10^ Tazemetostat is effective clinically for a portion of patients and provides an actionable therapeutic option for patients with significant unmet clinical need, yet the biological basis for variable responses remains unknown and the rationale for drug combinations is undefined.

Previous studies have shown that loss of tumour suppressor gene *SMARCB1* is the most common mutation in EPS, occurring in up to 93% of EPS cases.^6^, ^11^ While loss of *SMARCB1* is believed to play a crucial role in the pathogenesis of EPS, restoration of this protein‐coding gene does not solely stop the progression of disease.^12^ Thus, other signalling pathway mutations likely contribute to the disease and pathogenesis is a result of a complex genetic landscape.^6^ Targeting a single signalling pathway has conventionally proven insufficient, conceivably due to crosstalk between diverse biological pathways,^12^, ^13^ suggesting that therapies, which simultaneously target multiple biological signalling pathways are likely needed to improve patient outcomes. In this study, we have performed drug screening in combination with next‐generation sequencing (i.e. functional genomics) to define patient specific and disease‐wide therapeutic vulnerabilities, that is, ideally tazemetostat combination therapies. While this comprehensive approach did not yield a pan‐EPS drug combination for immediate clinical investigation, we have elucidated the molecular and functional characteristics of the two distinct EPS subtypes as a foundation for broader study.

## 2 METHODS AND MATERIALS

### 2.1 Cell lines

HS‐ES‐1, HS‐ES‐2R and HS‐ES‐2M were purchased from Riken (RCB2364, RCB2361, RCB2360, respectively, Riken, Japan) and maintained in DMEM supplemented with 10% foetal bovine serum (FBS) and 1% penicillin‐streptomycin (PS). VA‐ES‐BJ was purchased from ATCC (CRL‐2138, ATCC, Manassas, VA, USA) and also maintained in DMEM supplemented with 10% FBS and 1% PS. ESX, developed and provided by T. Tsukahara (Japan),^23^ was maintained in IMDM supplemented with 10% heat‐inactivated FBS and 1% PS. YCUS‐5 was developed and provided by co‐author Hiroaki Goto (Japan)^24^ and maintained in RPMI 1640 with 10% FBS and 1% PS. Epi‐544 was developed and provided by co‐author Keila E. Torres (Houston, TX, USA) and maintained in DMEM supplemented with 10% FBS and 1% PS. The BT‐12 cell line was provided by the Children's Oncology Group (COG) and maintained in Iscove's Modified Dulbecco's Medium (IMDM) plus 20% FBS, 4mM L‐Glutamine, 5 μg/ml insulin, 5 μg/ml transferrin and 5 ng/ml selenous acid and 1% PS. The G‐401 cell line was purchased from ATCC (CRL‐1441, ATCC) and cultured in McCoy's 5A medium supplemented with 10% FBS and 1% PS. HEK293 cells were also purchased from ATCC (CRL‐1573, ATCC) and cultured in RPMI with 10% FBS and 1% PS. All cell lines were grown at 37°C and 5% CO_2_. All cell cultures were tested to ensure mycoplasma negativity and authenticated by short tandem repeat analysis (University of Arizona Genetics Core, Table S4) using the Promega PowerPlex16HS Assay (Madison, WI, USA).

### 2.2 Primary cell culture generation

The EPS primary cell culture CF‐00442‐2 was received through the CuReFAST tumour bank program at the Children's Cancer Therapy Development Institute (cc‐TDI.org). Tumour tissue was minced and processed using a gentleMacs tissue dissociator and associated tumour dissociation kit (130‐093‐235 and 130‐095‐929, respectively, Miltenyi Biotec, Germany) per manufacturer's instructions. The resultant culture was grown in DMEM supplemented with 20% FBS and 1% (PS). The EPS primary cell culture PCB490‐5 was generated as described previously^6^ and maintained in RPMI 1640 with 10% FBS and 1% PS. All tissue samples for primary cell culture development were collected with patient consent (Advarra, protocol # cc‐TDI‐IRB‐1).

### 2.3 EPS tissue samples

Eps samples were gathered using the CuReFAST Biobank with informed consent obtained from patients and families. Co‐author Paul Huang and Robin L. Jones from the Royal Marsden Institute and Institute of Cancer Research supplied the tissue for CF‐01427 through CF‐01439. Co‐author Thomas G. P. Grünewald supplied the data for Eps_1 through Eps_11.

### 2.4 Therapeutic compound screen

The following compounds were purchased from (MedChemExpress Monmouth Junction, NJ, USA): CCT251545, MTX‐211, CUDC305, CUDC427, AMG232, ML329 and fasudil. Both 666‐15 and GSK‐J4 were purchased from R&D Systems (Minneapolis, MN, USA). Larotrectinib was purchased from Abmole (Houston, TX, USA). All other agents were purchased from Selleckchem (Houston, TX, USA). All agents were received as a dried powder and were reconstituted in DMSO (D8418, Sigma Aldrich, St. Louis, MO, USA) to a concentration of 10 mM or lower based on solubility specifications per manufacturer. Agents were plated at four concentrations (2, 0.2, 0.02 and 0.002 μM) in triplicate in a 384‐well format using the HP Tecan D300e and Perkin Elmer SciClone G3 digital dispensers. Cells were grown in T‐75 flasks until 80% confluent, trypsinised and plated at a density of 2000–2500 cells per well and incubated at 37°C with humidified 5% CO_2_ for 72 h. Cell viability was measured using CellTiter‐Glo 2.0^®^ (G9243, Promega, Madison, WI, USA) per manufacturer's instructions. Luminescence was measured using a BioTek Synergy HT plate reader (BioTek, Winooski, VT). IC_50_ values were determined using a nonlinear best fit method.

### 2.5 PABPC1 RNA interference

PABPC1 was silenced using shRNA kit from Dharmacon (L‐019598‐00‐0005, Lafayette, CO, USA) according to manufacturer instructions. PCB‐00490‐5 was cultured and seeded into a 384‐well dish and incubated for 24 h. The cells were then transfected and incubated for an additional 24 h. The 384‐well plate was then dosed using the Tecan D300e with Selinexor and HCQ and incubated for 72 h. The plate was then imaged using CellTiter‐Glo 2.0 according to manufacturer instructions.

### 2.6 Tazemetostat pretreatment combination therapy

VA‐ES‐BJ was cultured in growth media with tazemetostat at 300 nM for 144 h. The pretreated cells were seeded into half of a 384 multi‐well plate with growth media dosed with tazemetostat and untreated cells were seeded into the other half in growth media and incubated for 24 h. Plates was dosed with (+)‐JQ1 and UNC0642 at a concentration range of 0.01‐10 μM or with caffeine and theophylline at a concentration range of 0.005–100 μM and incubated for 72 h and imaged using CellTiter‐Glo 2.0.

### 2.7 BRD7/9 inhibition

VZ185 degrader and its control were obtained from Boehringer Ingelheim onpME Portal (Ingelheim, Germany). BRD7/9 protein levels were monitored in cells treated with VZ185 and its non‐degrading control cis‐VZ185 at 10 and 1000 nM. There was significant degradation in BRD9 but not in BRD7 as shown in Figure S11B. Inhibition curves were done using VZ185 on cell lines VA‐ES‐BJ and PCB‐490‐5; however, the IC_50_s were found to be in the tens of micromolars and the degrader molecule was not further pursued.

### 2.8 DNA and RNA extraction and sequencing

Material for the generation of whole exome and RNA sequencing data was isolated from all EPS cell lines as well as all FFPE tissue specimens. Each cell line was grown to 80% confluency, trypsinised and snap frozen. RNA and DNA were extracted and sequenced by Beijing Genomics Institute (BGI, San Jose, CA). The quality of DNA prior to extraction was adequate for each cell line (DNA fragment ≥ 250 bp), as well as the quality of RNA (DV < 200%). HiSeq 4000 was used for paired‐end sequencing with 40 million reads for RNA and 100× coverage for tumour DNA.

### 2.9 Whole exome and whole transcriptome sequencing analysis

Raw FASTQ sequencing files were run through our in‐house computational pipeline. Somatic mutations, variations and indels were called using Genome Analysis Toolkit (GATK) Version 4.0 and the GRCh38 human reference genome. Gene copy number variations were quantified as a log ratio of tumour copy to normal copy using Samtools and VarScan2. RNA sequencing data was analysed for gene expression and gene fusion events. Transcriptome data were aligned to STAR‐derived human transcriptome from GRCh38 human reference genome. Normalised gene expression was quantified using STAR aligner with RSEM.

### 2.10 Eigengene analysis of proximal versus distal epithelioid sarcoma

The top 5000 most variable genes in terms of expression in the first *n* samples were determined using standard variance and then clustered into co‐expression modules using the WGCNA package in R 3.4.1.^25^, ^26^ After clustering, sets of co‐expressed genes were annotated for functional overrepresentation using the DAVID web service, version 6.8.^27^ Functional overrepresentation was determined using the false discovery rate.^28^ For visualisation, the eigengene or central tendency of each module was plotted in heatmap form and used to cluster samples. After the original clustering, additional samples were clustered using the module members determined in the initial analysis, but the modules were not recalculated based on these new members.

### 2.11 Supervised learning analysis of proximal versus distal epithelioid sarcoma

The supervised machine learning approach to identify differential gene features is adapted from the PTM‐Biomarker analysis platform.^29^ In brief, post‐analysis whole exome and whole transcriptome sequencing data were ingested into the learning framework, which generates Boolean gene feature relationships differentiating proximal versus distal EPS. Top prioritised gene features were merged to create a joint heatmap of differential gene features linked by common biological roles as defined by interaction network and gene ontology analyses.

### 2.12 Western blot analysis

All cell samples were extracted in RIPA lysis buffer supplemented with complete protease inhibitor and phosphatase inhibitor cocktail (Thermo Fisher Scientific, Waltham, MA; #89901 and #78441, respectively). After incubation on ice for 30 min, samples were centrifuged at 12 000 × *g* for 5 min (at 4˚C), and supernatant was collected. Protein was quantified using the Pierce BCA assay kit (Thermo Fisher Scientific, Waltham, MA; #23224). Fifty micrograms of protein from each sample was loaded and separated in 7.5% SDS‐PAGE gel and transferred onto a 0.2 mm PVDF membrane using wet transfer method (90V for 90 min). The PVDF membrane was incubated with primary mouse anti‐BAF47 (SMARCB1) antibody (BD Transduction Laboratories, #612110) at a dilution of 1:500 in non‐fat powdered milk and tris buffered saline with tween (TBST), then placed on a rocker overnight at 4°C. For the BRD7/9 western blot anti‐BRD7 (Bethyl Labs, #A302‐304‐M) and anti‐BRD9 (Bethyl Labs, #A303‐781A) were used and thePABCP1 western blot used anti‐PABP (Abcam, ab153930). The membrane was then incubated with peroxidase labelled anti‐mouse IgG secondary antibody (Vector Laboratories, Burlingame, CA; #BA9200) at a dilution of 1:5000 and placed on a rocker for 1 h prior to protein visualisation. Following primary antibody visualisation, the PVDF membrane was incubated with primary rabbit anti‐GAPDH antibody (Cell Signaling Technology, Danvers, MA; #14C10) for the SMARCB1 and PABPC1 western blot, which was used as a positive control, at a dilution of 1:5000 then placed on a rocker for 1‐h prior. β‐actin (Abcam, #ab8227) was used for the BRD7/9 western blot with the same dilutions. The membrane was then incubated with peroxidase labelled anti‐rabbit IgG secondary antibody (Vector Laboratories, Burlingame, CA; #PI‐1000) diluted 1:5000 for 1 h at room temperature with gentle rocking prior to visualisation. All proteins were detected using enhanced chemiluminescence (ECL) and read on an IVIS Lumina Imaging System, with an exposure time of 5–10 s.

### 2.13 ICC staining

A Fisher brand premium glass cover slip (Fisher Scientific #12‐548‐BP) was placed into each well of a 6‐well plate. A total of 300 000 cells of each cell line were plated with 1.5 ml of appropriate media into each well, covering the glass cover slip. After 24 h, the cells were 50%–80% confluent. Cells were washed with PBS, then fixed with ice cold 100% MEOH for 5 min. Cells were washed three times with cold PBS, then blocked with 1% BSA in PBST (PBS+0.1% Tween20) for 30 min. Cells on the coverslip were then placed face up on parafilm and 100 μl of 1:100 BD Biosciences mouse anti‐Baf47 (SMARCB1) (612110) in blocking buffer was applied to each coverslip. Parafilm, coverslip and antibody were then covered with tinfoil to shield from light and allowed to incubate for 2 h at room temperature. After incubation, antibody was removed, and coverslips were placed into 6‐well dishes and washed three times with PBS. For secondary antibody treatment, cover slips were removed from the 6‐well plates and once again placed face up on parafilm. 150 μl of 1:1000 mouse Alexa fluor 488 (Invitrogen #A32723TR, Carlsbad, CA, USA) in blocking buffer was applied to each coverslip, then covered and allowed to incubate for 1 h at room temperature. Secondary antibody was then removed, and cover slips were placed into 6‐well dishes and washed three times with PBS. One drop of Vectashield antifade mounting medium with DAPI (Vector laboratories #H‐1200) was applied to each glass slide and the cover slip was placed on it with cells facing down. Nail polish was used to fix coverslip to slide. Slides were imaged using a LSM800 confocal inverted laser scanning microscope (Zeiss, Oberkochen, Germany).

### 2.14 Immunohistochemistry

**FIGURE 1:**
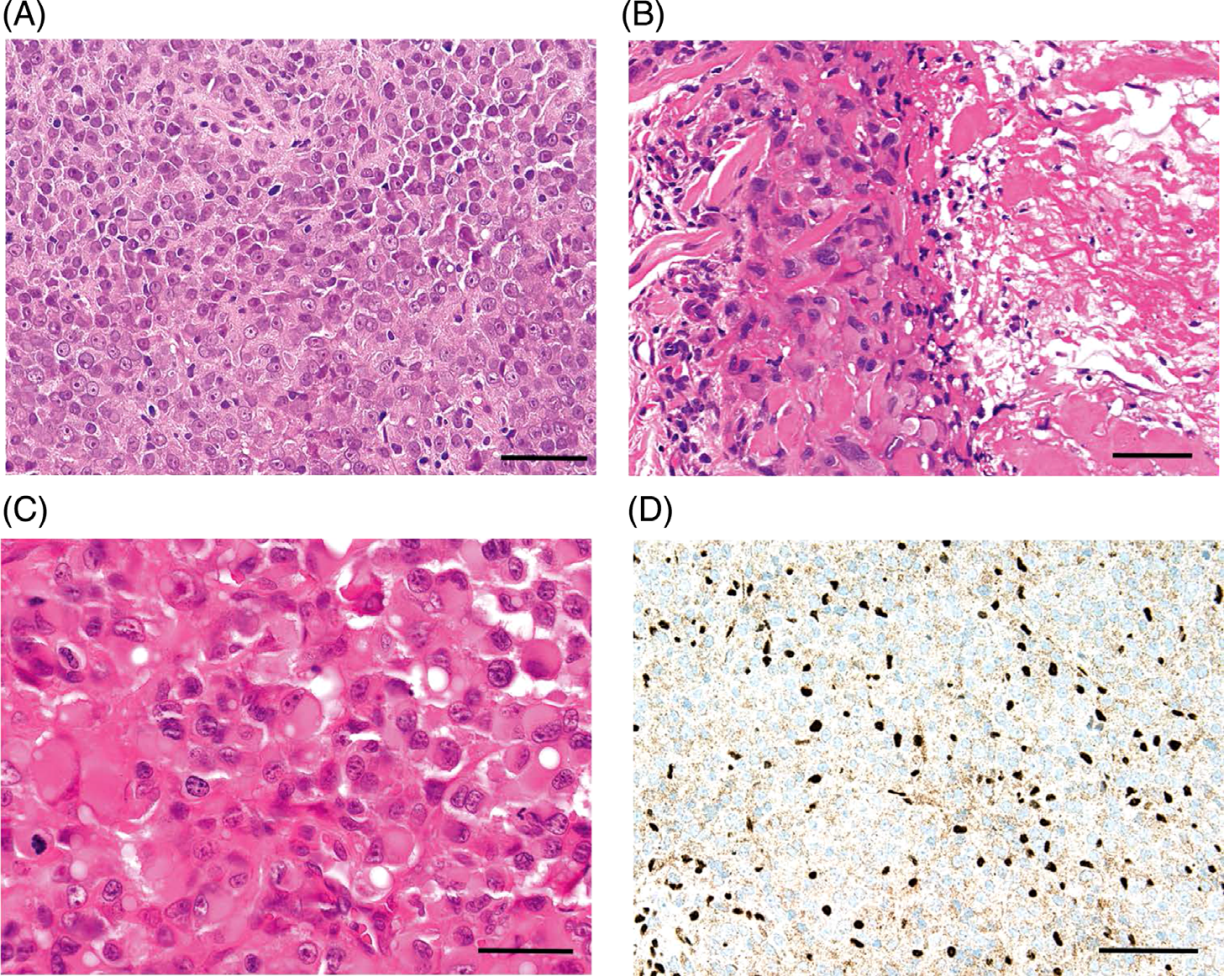
Proximal versus distal histology. (A) Conventional‐type/distal‐type epithelioid sarcoma. The tumour is composed of sheets of essentially uniform and relatively small to medium‐sized cells with rounded to ovoid vesicular nuclei, prominent nuclei and moderate amounts of eosinophilic cytoplasm. The cells have a somewhat histiocytoid‐appearance, and cellular atypia is minimal. A mild chronic inflammatory infiltrate, largely of small lymphocytes, is intermingled (haematoxylin and eosin, ×100). Scale bar, 100 μm. (B) Conventional‐type/distal‐type epithelioid sarcoma. Nests and sheets of epithelioid cells, here with abundant eosinophilic cytoplasm, are present adjacent to a large area of fibrinoid material/incipient geographic tumour necrosis (right of field) (haematoxylin and eosin, ×200). Scale bar, 50 μm. (C) Proximal‐type epithelioid sarcoma. This tumour is composed of monotonous sheets of large, epithelioid and polygonal cells with ovoid vesicular nuclei and prominent, sometimes multiple nucleoli and abundant, palely eosinophilic cytoplasm. In many cells, the combination of extensive eosinophilic cytoplasm and eccentrically oriented nuclei give the cells marked rhabdoid appearances (haematoxylin and eosin, ×400). Scale bar, 25 μm. (D) Conventional‐type/distal‐type epithelioid sarcoma. Immunohistochemistry for INI1 (SMARCB1). This protein is absent in nuclei in approximately 90% of both classic‐type and proximal‐type epithelioid sarcoma, and here, the lesional nuclei show diffuse absence of expression of INI1. This is in contrast to the surrounding smaller numbers of lymphocytes and local stromal and endothelial cells, which show strong expression of the intact protein in their nuclei (immunoperoxidase, ×200). Scale bar, 50 μm

In Figure 1 deparaffinised sections of each tumour were stained with anti‐INI1/SMARCB1 antibody (anti‐BAF47) mouse monoclonal antibody (Sigma‐Aldrich, Burlington MA), using heat‐induced epitope retrieval. Appropriate positive and negative controls were used throughout.

### 2.15 Microsatellite instability analysis

Microsatellite instability (MSI) PCR testing was performed by LabCorp Research Services (Burlington, NC, USA) using the MSI Analysis System v1.2 (MD1641; Promega, Madison, WI, USA). Fluorescently labelled primers were used for co‐amplification of five mononucleotide repeat markers (BAT‐25, BAT‐26, NR‐21, NR‐24 and MONO‐27) to detect MSI, as well as two pentanucleotide repeat markers (Penta C and Penta D) included to confirm sample identity. The PCR products were sized with an Applied Biosystems 3500 xL Genetic Analyzer (Invitrogen) and instability was defined as heterozygosity or allele size shifts compared to the associated normal reference tissue sample. Tumour samples were categorised as MSI‐High if two or more loci demonstrated instability, MSI‐Low if one locus demonstrated instability, and stable if all loci matched between the tumour sample and its normal reference.

For tumour samples, which lacked an available normal reference sample, instability was defined as a 3 bp shift or greater from the common allele sizes of the five quasi‐monomorphic markers (NR‐21 = 98 bp, BAT‐26 = 113 bp, BAT‐25 = 120 bp, NR‐24 = 130 bp, MONO‐27 = 150 bp) as described by Bacher et al.^30^ Given that instability in the form of 1 bp shifts cannot be appreciated without the direct normal comparison, samples which showed no apparent instability by this unpaired method of analysis were categorised as ‘No MSI detected’ rather than certainly stable.

Msisensor2 (available at https://github.com/niu‐lab/msisensor2), an updated version of MSIsensor,^31^ was used in order to determine MSI status. The tool outputs an MSI score, which is the percentage of all valid sites – defined as sites with sequencing coverage over a user defined threshold – that were classified as MSI by a machine learning model. The developers recommend calling a sample as MSI‐H if the MSI score is above 20%. We validated the results generated by the tool by comparing them against a subset of samples where MSI was determined with support from LabCorp. Specifically, for samples where we had matched tumour‐normal pairs, MSI‐PCR was used. In cases with just tumour only samples, the pentaplex PCR panel was used and marker length compared to allele frequency tables.

### 2.16 Tumour mutational burden

High/modifier (HM) and low/moderate (LM) mutation VCFs were used for all samples. Non‐silent mutations were denoted as all SNPs in the HM mutation results, whereas silent were denoted as all the SNPs in the LM mutation results. Indels were denoted as all mutations that had an insertion or deletion greater than ‘2 nucleotides’ among both HM and LM mutations; however, the majority of InDels came from HM mutations. A stacked bar‐graph was then created using the silent, non‐silent and InDels in every patient using the ‘Seaborn’ python package.

### 2.17 Copy number variation

All biopsy and cell‐line data were subset as Distal or Proximal. An average of the exponential version of the log_ratio gain/loss data was computed across samples for the cytogenetic bands and these bands were sorted based on their position in the chromosome from 1 through 23, X and Y. A bar graph was created with the processed input using the ‘Seaborn’ python package.

### 2.18 Statistical analysis

All statistical analyses were performed with GraphPad Prism V9.3.1. Low, Mid and High in Tables 1 and 2 were determined using one‐sided Student's *t*‐test performed in Microsoft Excel, with Mid representing no significant difference between the expression in the sample and the expression in the GTExII data set, low representing statistically low expression, and high representing statistically high expression. Statistical tests used are described for each result presented.

**TABLE 1:** Procured epithelioid sarcoma cell line resources

							***SMARCB1* status this report**				
**Cell Line/culture name**	**Age (years)**	**Sex**	**Location**	**Primary mutation; secondary mutation(s)**	**Year reported**	**Cell line/culture source**	**Protein status**	**Mutation status**	**CNV status**	**RNA status**	**Ref**
HS‐ES‐1 (CF‐00775)	60	M	Primary, right perineum	SMARCB1– +vimentin, +cytokeratin, +EMA/MUC1	1997	Sonobe (Riken, Japan)	Null	N/A	Hom intragenic loss (3344 bp)	Low (0×)	^32^
HS‐ES‐2M (CF‐00776)	‐	M	Lung mets (2m)	SMARCB1–	1993	Sonobe (Riken, Japan)	Null	WT	Het gene loss	Low (0.14×)	^12^
HS‐ES‐2R (CF‐00777)			EPS recurrence (2r)								
			Primary unknown								
YCUS‐5 (CF‐00748)	3	F	Primary, neck	SMARCB1–	1999	Nagashima (Yokohama City University School of Medicine)	Null	WT	Het gene loss	Low (0.21×)	^24^
				+cytokeratin, +EMA/MUC1, +vimentin, +ALK, +NSE, +TH							
VA‐ES‐BJ (CF‐00750)	41	M	Bone marrow mets	SMARCB1–/–	1995	Helson (St. Agnes Hospital)	Null	WT	Diploid	Low (0.53×)	^33^
			Primary, vertebral	+AE1/AE3, +vimentin, +EMA/MUC1							
PCB‐00490‐5	22	F	Primary, shoulder	SMARCB1–	2018	Keller (Children's Cancer Therapy Development Institute)	Null	N/A	Het gene loss, Hom intragenic loss (100 bp)	Mid (1.07×)	^8^
				+ABL1, +NOTCH1, +MDM4, +PAK4, +MAP4K5							
CF‐00442‐2	26	M	Pulmonary mets Primary, right hand	SMARCB1–	2019	Keller	Trace	Gene fusion	Het gene loss	High (2.57×)	N/A
Epi‐544 (CF‐00979)	‐	‐	Primary, foot	SMARCB1–	2011	Lev (MD Anderson Cancer Center)	Trace	WT	Diploid	High (3.16×)	^34^
				0							
ESX (CF‐02018)	73	F	Primary, left thigh	Hetero SMARCB1–	2013	Sato (Sapporo Medical University School of Medicine)	Present	Inframe deletion (c.10_12 delATG)	Diploid	High (8×)	^23^
				+AE1/AE3, +vimentin S100–, CD34–, CA125–							
PCB495	‐	M	‐	SMARCB1–	2018	Keller	‐	Splice acceptor variant (c.629‐1G>A)	Hom intragenic loss (425 bp)	‐	^8^
SJSTS046147_X1	18	M	Primary, spine	Not reported	Unknown	St. Jude's Children's Research Hospital	‐	‐	‐	‐	N/A
CF‐01311	37	F	Primary, vulva	Not reported	2019	Keller	Trace	‐	‐	‐	N/A

*Note*: RNA status factor is ratio between tumour expression and median normal skeletal muscle expression from GTExII cohort (median value 25.5 TPM). Low represents statistically low expression, High represents statistically high expression, and Mid representing no significant difference between the expression in the sample and the expression in the GTExII data set.

Hom = homozygous, Het = heterozygous, ‐ = data not available.

**TABLE 2:** Summary of epithelioid sarcoma deidentified patient data

						*SMARCB1* status this report		
Sample ID	**Age (years)**	Sex	Location	Clinical SMARCB1 status	EPS tumour type	**Mutation status**	**CNV status**	**RNA status**
CF‐00442	26	M	Right arm, metastatic	SMARCB1–	Distal	Potential LOF fusion	Het gene loss	High (2.57×)
CF‐00463	29	M	Vertebral primary, multiple locations metastatic	SMARCB1–	Proximal	WT	Het gene loss	Low (0.26×)
CF‐00477	30	M	Left arm primary	SMARCB1–	Distal	Cancer Predisposition Mut (c.897G>A)	Diploid	High (1.49×)
CF‐00488	15	M	Right thumb primary	SMARCB1–	Distal	Cancer Predisposition Mut (c.897G>A)	Diploid	‐
CF‐00490	42	M	‐	‐	‐	‐	‐	Low (0.49×)
CF‐00982	15	F	Right thumb primary	SMARCB1–	Distal	N/A	Het gene loss; Hom intragenic loss (328 bp)	Mid (1.07×)
CF‐00983	8	M	Left hand finger primary	SMARCB1–	Distal	N/A	Het gene loss; Hom intragenic loss (200 bp)	High (1.6×)
CF‐01427	50	M	Buttock	‐	Proximal	LOF Mut (c.510_515delTTCCGC)	Diploid	Low (0.62×)
CF‐01428	33	M	Thigh	‐	Distal	Disruptive INDEL (c.829_830insTCC)	Diploid	High (2.46×)
CF‐01429	35	M	Perineum	‐	Proximal	Disruptive Frameshift (c.222_223insCGTG)	Het gene loss; Hom intragenic loss (266 bp)	Low (0.43)
CF‐01430	18	M	Groin	‐	Proximal	Missense Mut (c.673G>A)	Het gene loss; Hom intragenic loss (66 bp)	Low (0.64×)
CF‐01431	64	M	Shoulder		Proximal	LOF Mut (c.508_509insGAGA)	Het gene loss	High (2.11×)
CF‐01432	29	F	Forearm	‐	Distal	LOF Mut (c.219A>C)	Diploid	High (1.82×)
CF‐01433	49	F	Shoulder	‐	Proximal	WT	Diploid	High (2.02×)
CF‐01434	28	M	Arm	p.Leu297_Gly302del p.Leu306_Gly311del p.Leu251_Gly256del p.Leu288_Gly293del	Distal	Disruptive Frameshift (c.445_457delACAACCATCAACA)	Het gene loss	Low (0.34×)
CF‐01435	73	F	Left labia majorus	‐	Proximal	N/A	Hom gene loss; Hom intragenic loss (128 bp)	Low (0.65×)
CF‐01436	37	M	Thigh	‐	Distal	WT	Diploid	High (1.93×)
CF‐01437	77	F	Perineum	‐	Proximal	N/A	Hom gene loss; Hom intragenic loss (97 bp)	Low (0.33×)
CF‐01438	40	M	Thigh	‐	Distal	Disruptive Frameshift (c.306_307delCA)	Diploid	Mid (1.01×)
CF‐01439	32	F	Thigh		Distal	LOF Mut (c.219A>C)	Diploid	High (2.66×)
CF‐01425	39	F	Vulva		Proximal	‐	‐	‐

*Note*: RNA status factor is ratio between tumour expression and median normal skeletal muscle expression from GTExII cohort (median value 25.5 TPM). Low represents statistically low expression, High represents statistically high expression, and Mid representing no significant difference between the expression in the sample and the expression in the GTExII data set.

Mut = mutation, Hom = homozygous, Het = heterozygous, LOF = loss of function, ‐ = data not available.

## 3 RESULTS

### 3.1 Molecular characterisation of *SMARCB1* status in EPS samples

**FIGURE 2:**
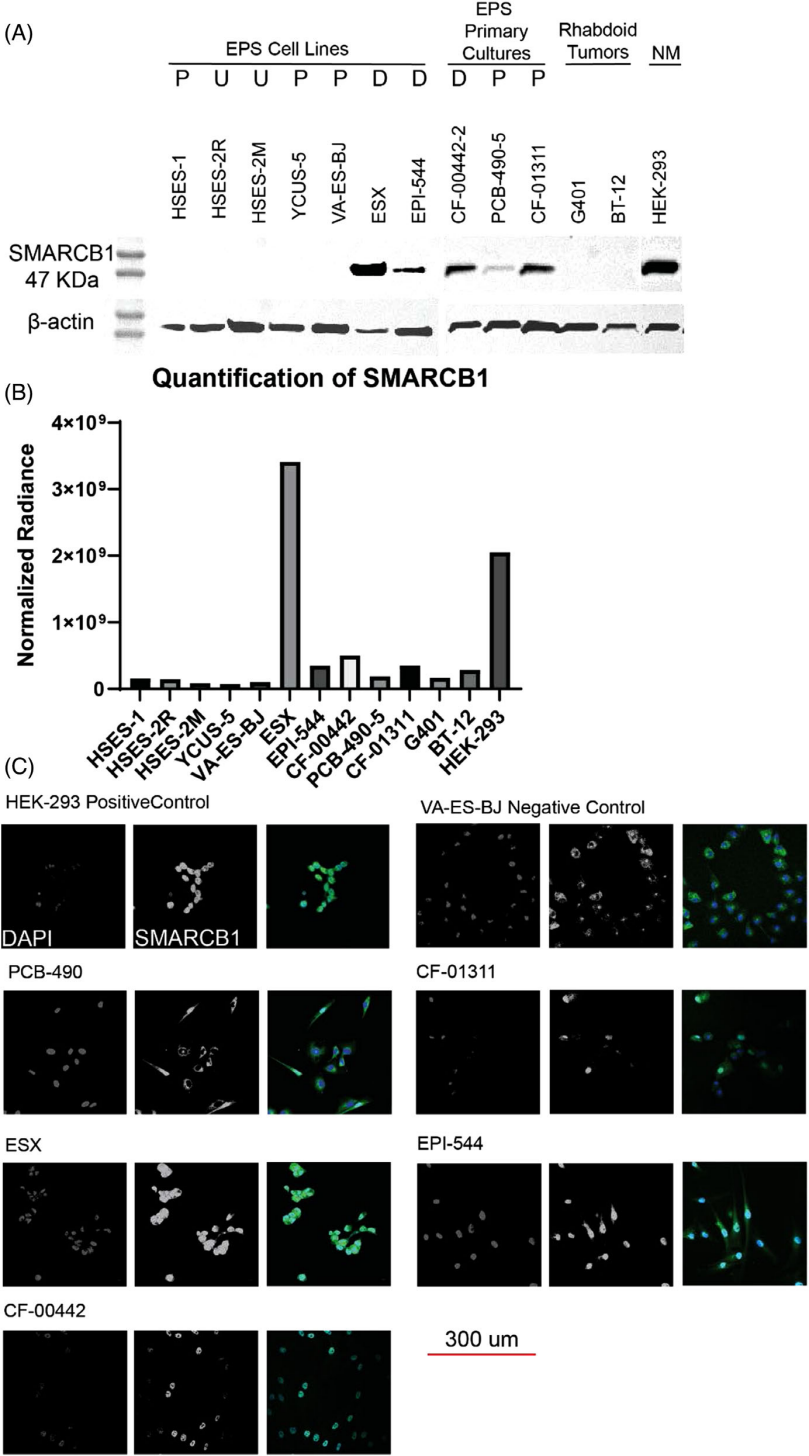
Protein analysis and immunocytochemistry of SMARCB1 in EPS cultures. (A) Immunoblot of SMARCB1. (B) The quantification of the previous panel with all samples normalised to HSES‐2M β‐actin. (C) SMARCB1 is labelled with Alexa fluor 488 (green), and the nucleus is stained with DAPI (blue). The scale bar is 300 μm and scale is the same for all of the cell lines. Nuclear staining of SMARCB1 is demonstrated in HEK‐293, ESX, CF‐00442 and EPI‐544, while absent or mostly absent in PCB‐490, VA‐ES‐BJ and CF‐01311. Alexa fluor 488‐positive staining of cellular cytoplasm is likely a result of non‐specific antibody binding given that SMARCB1 is considered to be a nuclear protein. P = proximal, D = distal, U = unknown, NM = normal. Proximal versus distal location is given as anatomical, not histological

**FIGURE 3:**
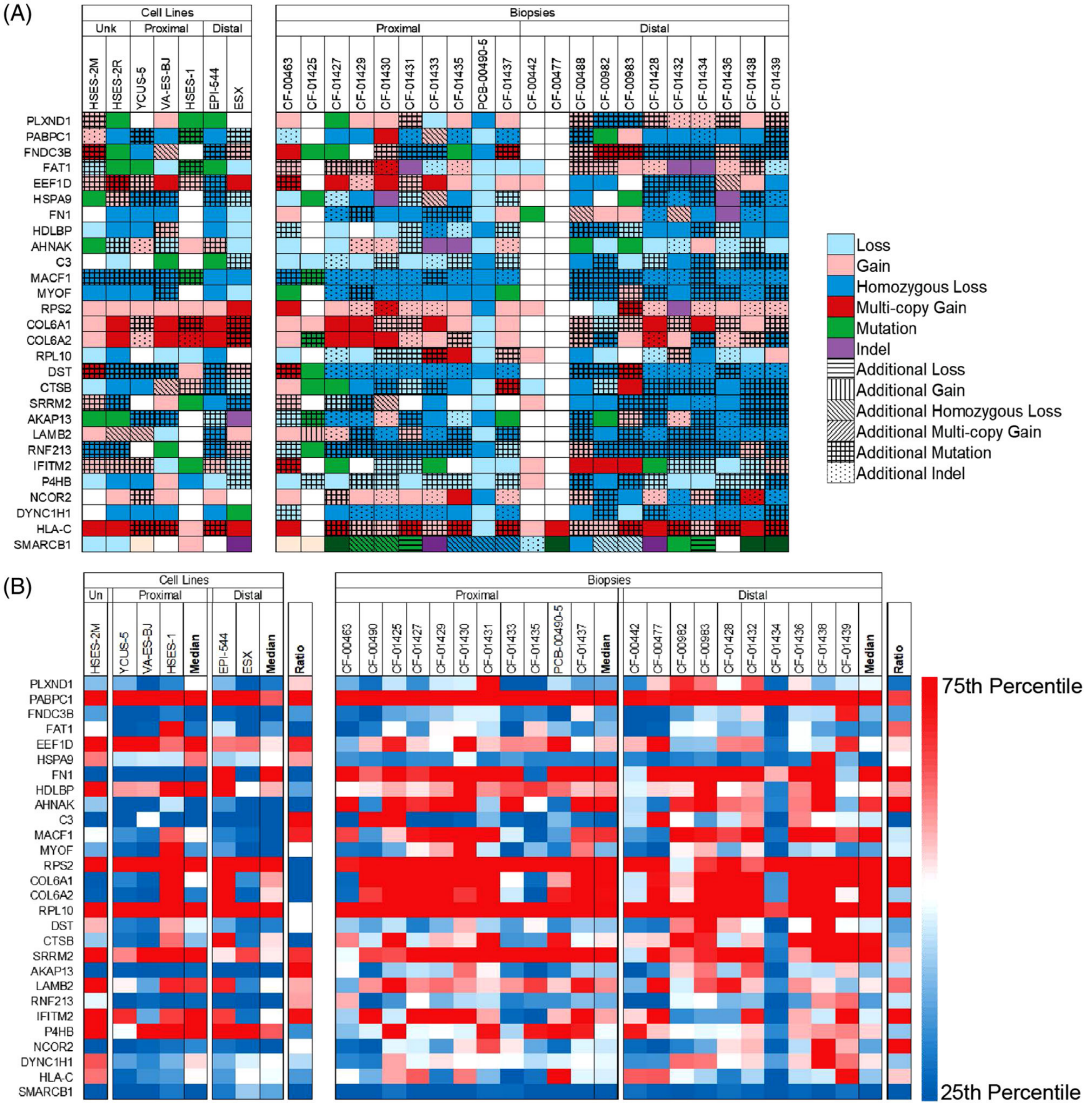
DNA and RNA expression. (A) Recurrent gene mutation, insertions/deletions or copy number changes prioritised by internal molecular analysis pipeline. (B) RNA expression of prioritised genes in panel A

For the distal and proximal subtypes of EPS (Figure 1A–D), we sought to collect and characterise all available biopsies/surgical resection samples and cell lines. Sources of biopsy tissues included a US‐based cohort (Keller Lab) and a Germany‐based cohort (Grünewald lab). To confirm *SMARCB1* status in publicly available EPS cell lines and primary culture resources, we performed protein studies of *SMARCB1* via western blot (Figure 2A, Tables 1 and 2) and compared expression against *SMARCB1* levels in *SMARCB1* wild type (WT) a normal cell line (HEK‐293) and *SMARCB1* null rhabdoid tumour cell lines (G401, BT‐12). Protein studies confirmed *SMARCB1* expression was absent or present only at trace levels in ten of eleven (10/11) EPS cell models tested. Only the ESX cell line demonstrated *SMARCB1* levels comparable to the *SMARCB1* WT normal cell lines. In EPS cell lines with *SMARCB1* expression we investigated expression and localisation of SMARCB1 in EPS cell models via immunocytochemistry (ICC) (Figure 2C), demonstrating no nuclear SMARCB1 expression in VA‐ES‐BJ or PCB‐490‐5, but retained nuclear SMARCB1 expression in CF‐01311, ESX, Epi‐544 and CF‐00442‐2, consistent with western blotting. To further define the *SMARCB1* status of EPS cell models and patient samples, we performed DNA whole exome and RNA whole transcriptome sequencing of the EPS sample cohort to identify pathogenic *SMARCB1* aberrations (Tables 1 and 2, Figure 3A, Tables S1 and S2). Identified aberrations include heterozygous gene loss events (three cell models, six patient samples), homozygous gene loss (one cell model, four patient samples), homozygous focal region loss (two cell models, six patient samples), reduced RNA expression (four cell models, eight patient samples) and disruptive genomic regions (three cell models, thirteen patient samples) (Tables 2 and 3). Across the EPS cohort, 7 cell models and 11 patient samples demonstrated strong evidence of SMARCB1 loss (null protein expression or evidence of homozygous inactivation of *SMARCB1*), 3 cell models and 9 patient samples demonstrated weak evidence of SMARCB1 loss (trace protein expression or evidence of heterozygous inactivation of *SMARCB1*) and 1 cell culture and 1 patient sample lacked evidence of *SMARCB1* alteration (Tables 1 and 2). Among published cell lines with nuclear SMARCB1 expression, ESX was found to have a heterozygous in frame deletion (p.Met4del), while no *SMARCB1* alterations were identified in Epi‐544.

**TABLE 3:** Focused compound selection (version 5)

Target	Drug name	Clinical status
RNA transcription initiation	Triptolide	Phase I
HDAC‐pan	Panobinostat*	Phase III
20S Proteasome	Bortezomib	Phase III
mTORC1,2	INK128/sapanisertib/tak228	Phase I/II
HDAC1/2/3/10, PI3K	Cudc907	Phase I/II
CDK1/2/4/6/9	At7519	Phase I/II
Proteasome	Ixazomib	Phase III
STAT 4	Napabucasin	Phase III
HSP90	Cudc305	Phase I
AKT, ERK	Onc201	Phase I/II
ALK, BMP	Ldn‐212854	preclinical
ATF1/CREB	666‐15	preclinical
ATR	Azd6738	Phase I/II
Beta‐blocker	Propranolol HCL	Phase I/II
B‐Raf	Vemurafenib	Phase I/II
BRD2,3,4 and BRD‐T	Gsk525762/i‐bet‐762	Phase I/II
BRD4, PI3k	Ly294002	preclinical
BRD4, PLK1, TAF1, TAF1L, CBP, P300	Bi2536	Phase I/II
CDC25a; iron chelation	Ciclopirox	FDA approved
CDK 4/6	Ribociclib	Phase III
CDK8/19, Wnt	Cct251545	preclinical
CENP‐E	Gsk923295	Phase I
CHK1/2	Prexasertib/LY2606368	Phase I/II
c‐MET, ALK	Crizotinib	Phase III
CPB, CREBBP/EP300	Sgc‐cbp30	preclinical
CRM‐1/XPO‐1	Selinexor	Phase III
EGFR (allosteric inhibitor)	Eai045	preclinical
EGFR‐PI3K	Mtx‐211	preclinical
EZH2	Tazemetostat	Phase I/II
FGFR1‐4	Ly2874455	Phase I
HDAC1/2, EGFR, HER2	Cudc101	Phase I
HDAC1/3	Entinostat	Phase III
IAP	Cudc427	preclinical
IGF1R	Bms‐754807	Phase I/II
IKBKE	Amlexanox	Phase II
JAK1/2	Ruxolitinib	Phase III
JMJD	Gsk‐j4	preclinical
JAK1/2, TBK, IKKε	Cyt387	Phase III
LSD1	Sp‐2509	preclinical
MCL‐1	S63845	preclinical
Mdm2	Amg232	Phase I/II
MEK1/2	Cobimetinib	Phase III
MITF	Ml329	preclinical
Mitochondrial energy metabolism	Cpi‐613	Phase III
mTOR	Sirolimus/rapamycin	Phase 4
mTOR, PI3K	BKM120/buparlisib	Phase I/II
Multiple RTK, PDGFR, SRC, EphB4	Dasatinib	Phase I/II
Multiple RTK, VEGFR, PDGFR, RAF	Sorafenib	Phase III
p300	C646	preclinical
PI3Ka/d, NFkB	Pictilisib	Phase I/II
PIM 1‐2‐3‐ Kinase	Azd1208	Phase I
Proton pump	Esomeprazole	FDA approved
ROCK	Fasudil	Phase III
S100A9; microenvironment	Tasquinimod	Phase III
S100B	Pentamidine	Phase I/II
TAR RNA‐binding protein 2	Enoxacin/penetrex	FDA approved
TNFa induction	Dmxaa	Phase III
TP53 (mutant)	Apr‐246	Phase III
pan‐TrkA/B/C, ROS1, ALK	Entrectinib	Phase II
TrkA, TrkB, TrkC	Larotrectinib	FDA approved
Wee1	MK‐1775/adavosertib	Phase I/II

* is used a a positive control.

### 3.2 Molecular status of EPS samples

Having confirmed SMARCB1 expression in EPS cell models, we expanded molecular characterisation to include DNA whole exome and RNA whole transcriptome sequencing of EPS cell lines, cell cultures and patient‐origin tumour tissues (Figure 3), identifying recurrent variations in a subset of 26 genes including *SMARCB1* (Figure 3A) and corresponding gene expression of the frequently altered genes (Figure 3B). Notable genomic DNA features included *PABC1* and *RPS2* stochastic gains (but uniformly high RNA expression), yet no consistent secondary genomic event was observed.

### 3.3 Eigengene analysis of proximal versus distal EPS

**FIGURE 4:**
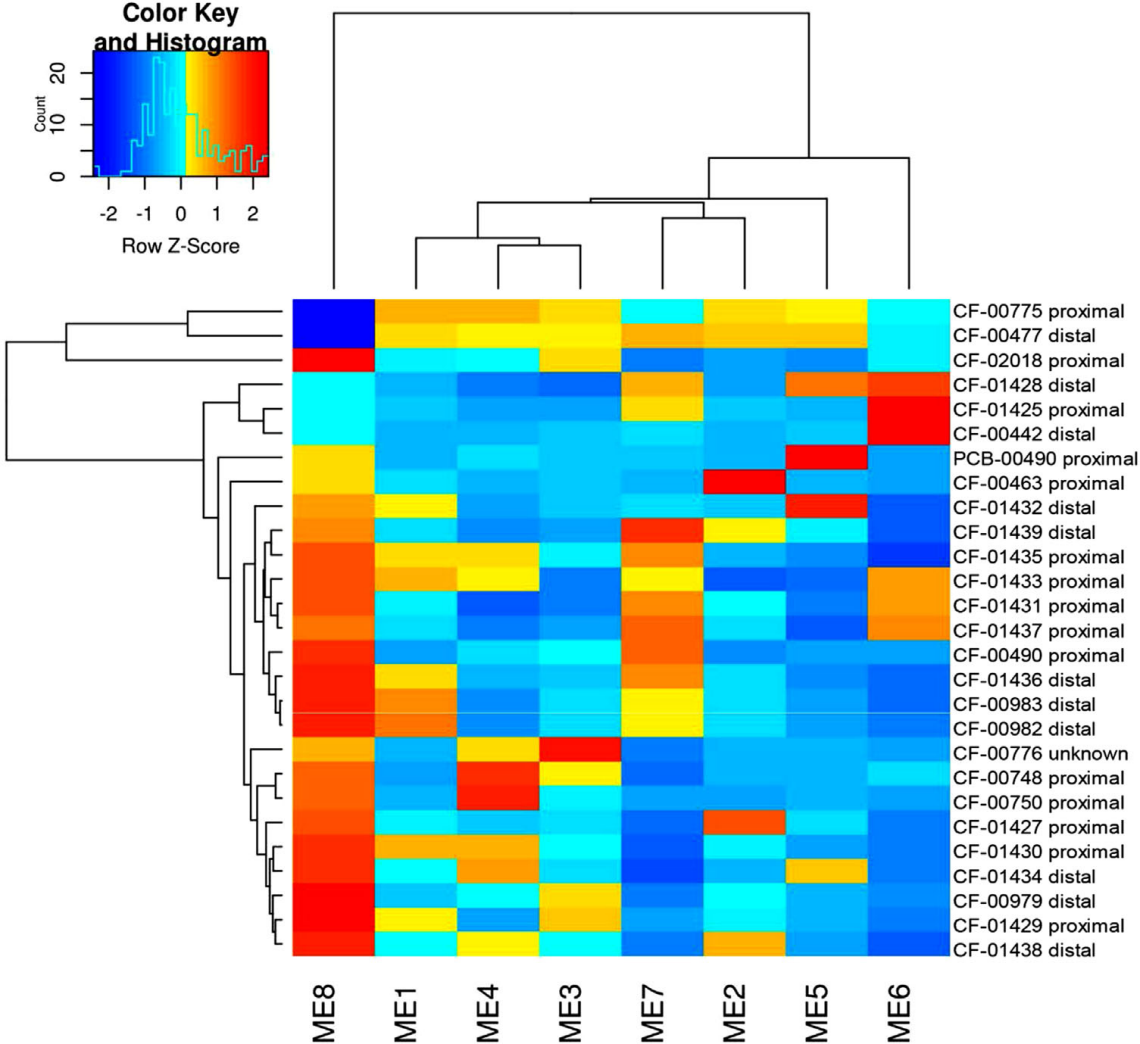
Eigengene proximal versus distal gene expression analysis. Eigengene modules showed enrichment for zinc finger and KRAB domain proteins in module 1; LDL receptors in module 2; DNA repair‐related processes, and hypermutation of immunoglobin genes in module 3; MYB‐related genes in module 4; cell cycle regulation, extracellular matrix and cell adhesion processes in module 6 and 7 and specific signalling pathways including GNRH signalling, oxytocin signalling, antigen processing, and presentation in module 5

We applied small‐cohort eigengene analysis to the EPS whole transcriptome sequencing data to identify functional gene modules differentially expressed between proximal and distal EPS subtypes (Figure 4). We identified potentially differentially regulated modules via unsupervised clustering of the eigengenes. DAVID analysis of the largest modules (from a total of 8) resulting from eigengene analysis were significantly enriched (FDR < 5%) for generic signalling and signal peptide ontology terms. Module 1 showed significant enrichment for Zinc finger and KRAB domain proteins, suggesting a possible role in or loss of function of epigenetic regulation.^14^ Module 2 showed notable but non‐significant (FDR ∼5%) enrichments for LDL receptor associated genes (Figure S1). Module 3 showed notable but non‐significant (FDR ∼11%) enrichments for DNA repair‐related terms and hypermutation of immunoglobin genes (GO:0016446). Module 4 showed significant enrichment for MYB‐related genes and suggestive enrichment for cell cycle regulation. Module 5 also showed significant or notable enrichment for a number of specific signalling pathways, including GNRH signalling, oxytocin signalling and antigen processing and presentation. Modules 6 and 7 were both enriched for extracellular matrix and cell adhesion terms, suggesting genes in these modules could be related to tumour microenvironment and adhesion differences between subtypes.

### 3.4 Supervised machine learning analysis of proximal versus distal EPS

**FIGURE 5:**
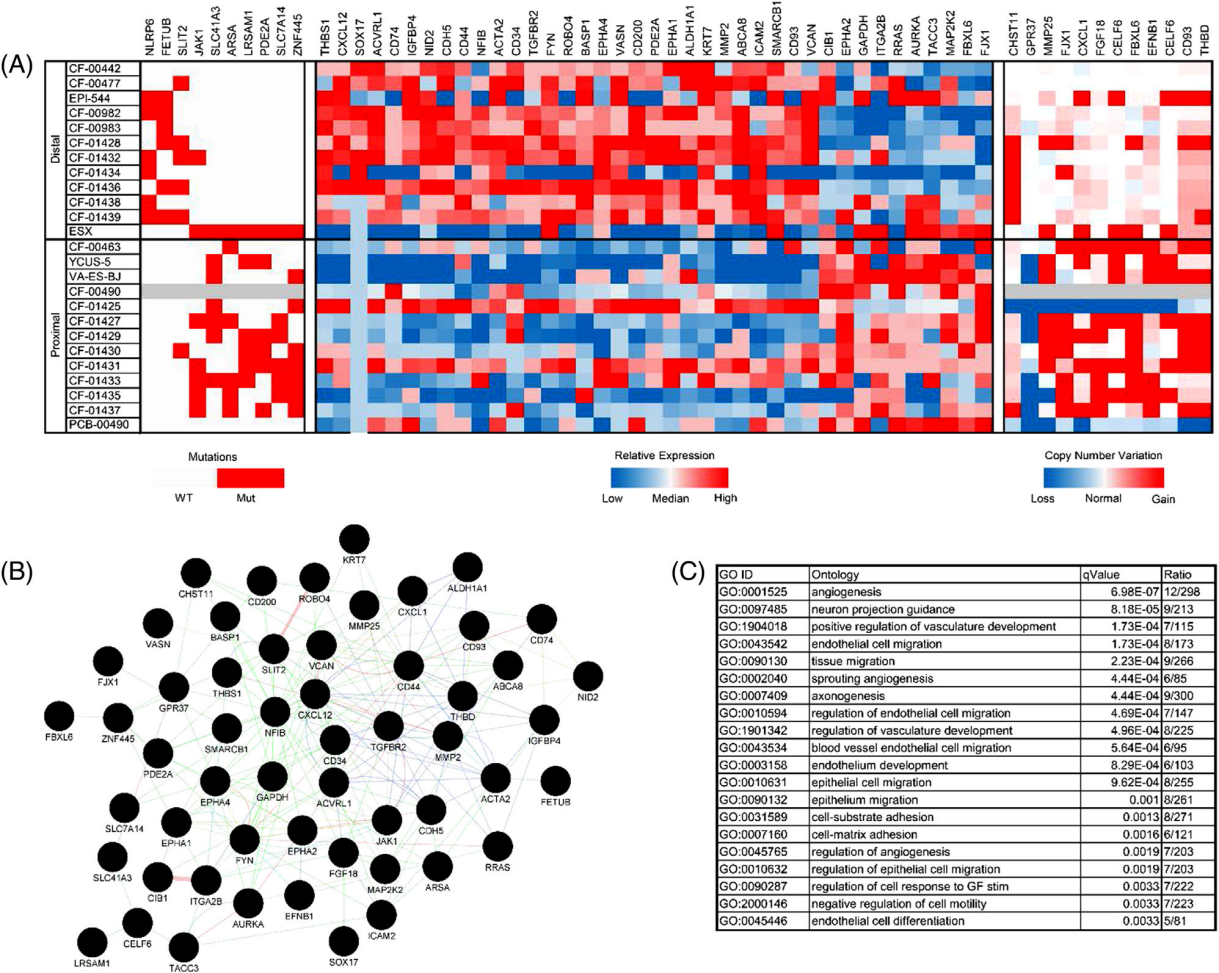
Proximal versus distal supervised learning analysis. Whole exome and whole transcriptome sequencing data from the EPS sample cohort were integrated in a supervised learning framework to identify molecular features differentiating proximal versus distal EPS subtypes with cohesive biological relevance. (A) Heatmap of mutation, copy number variation and gene expression features differentially occurring in proximal vs distal EPS samples. (B) Interaction and connection network of genes identified in differential EPS subtype analysis. (C) Gene ontology analysis of genes identified during differential analysis. The top 20 ontology classes are reported here. The full list is provided in Table S3. Note: CXCR4, the receptor for CXCL12, is the mechanistic target of plerixafor and BL‐8040

We applied a supervised machine learning approach to the whole exome and whole transcriptome sequencing data sets to identify gene features and associated biological processes differentially present in proximal versus distal EPS (Figure 5A). The genes prioritised in the heatmap define a connected network centred around *SMARCB1*, *NFIB*, *CXCL12*, *VCAN*, *TGFBR2*, *CD34* and *CD44* (Figure 5B). Ontology analysis of the prioritised genes identified ontology processes differentially present in distal versus proximal, which focus on angiogenesis‐associated processes, epithelial and epithelium migration and cell‐substrate and cell‐matrix adhesion (Figure 5C). Overall, results suggest a significant increase in angiogenesis‐associated activity and vasculature development in distal EPS (consistent with anecdotal clinical reports of response to pazopanib^15^, ^16^), as well as processes associated with cell migration and extracellular matrix activity.

Multiple biologically relevant genes were identified through the supervised learning analysis, including several genes significantly upregulated in distal versus proximal EPS (Figure S2) and significantly upregulated in proximal versus distal EPS (Figure S3), as well as genes with elevated expression in distal EPS (Figure S4) and proximal EPS (Figure S5). Top distal overexpressed genes include *NID2* (basement membrane glycoprotein with a role in extracellular matrix interactions, 7× overexpression), *KRT7* (keratin family member gene with intracellular roles, 17.8× overexpression), *FYN* (Src family proto‐oncogene known to be inhibited by dasatinib at clinically relevant concentrations, 3.3× overexpression), *SMARCB1* (3.1× overexpression), *CXCL12* (*CXCR4* cytokine ligand implicated in tumour growth and metastasis, 6.5× overexpression), *MMP2* (extracellular matrix protein, 7.4× overexpression), *BASP1* (membrane‐bound protein abundantly expressed in the brain, 2.5× overexpression) and *VASN* (binds and regulates *TGFβ*, 2.6× overexpression). Top proximal overexpressed genes include *SHC1* (Src homology‐containing adapter protein that couples activated growth factor receptors to signalling pathways, 2.8× overexpression), *C19orf33* (also called *H2RSP*, involved in single‐ and double‐stranded DNA binding, 6.5× overexpression), *RIPK4* (serine‐threonine kinase involved in *PKCδ* and *NF‐κB* signalling pathways, 12.2× overexpression), *EPHA2* (involved in numerous processes including cancer development and progression, 2.4× overexpression), *KRT8* and *KRT18* (*KRT8* and *KRT18* dimerise and are involved in signal transduction and cellular differentiation, 4.6× overexpression and 6.6× overexpression, respectively).

### 3.5 Gene expression in EPS samples

**FIGURE 6:**
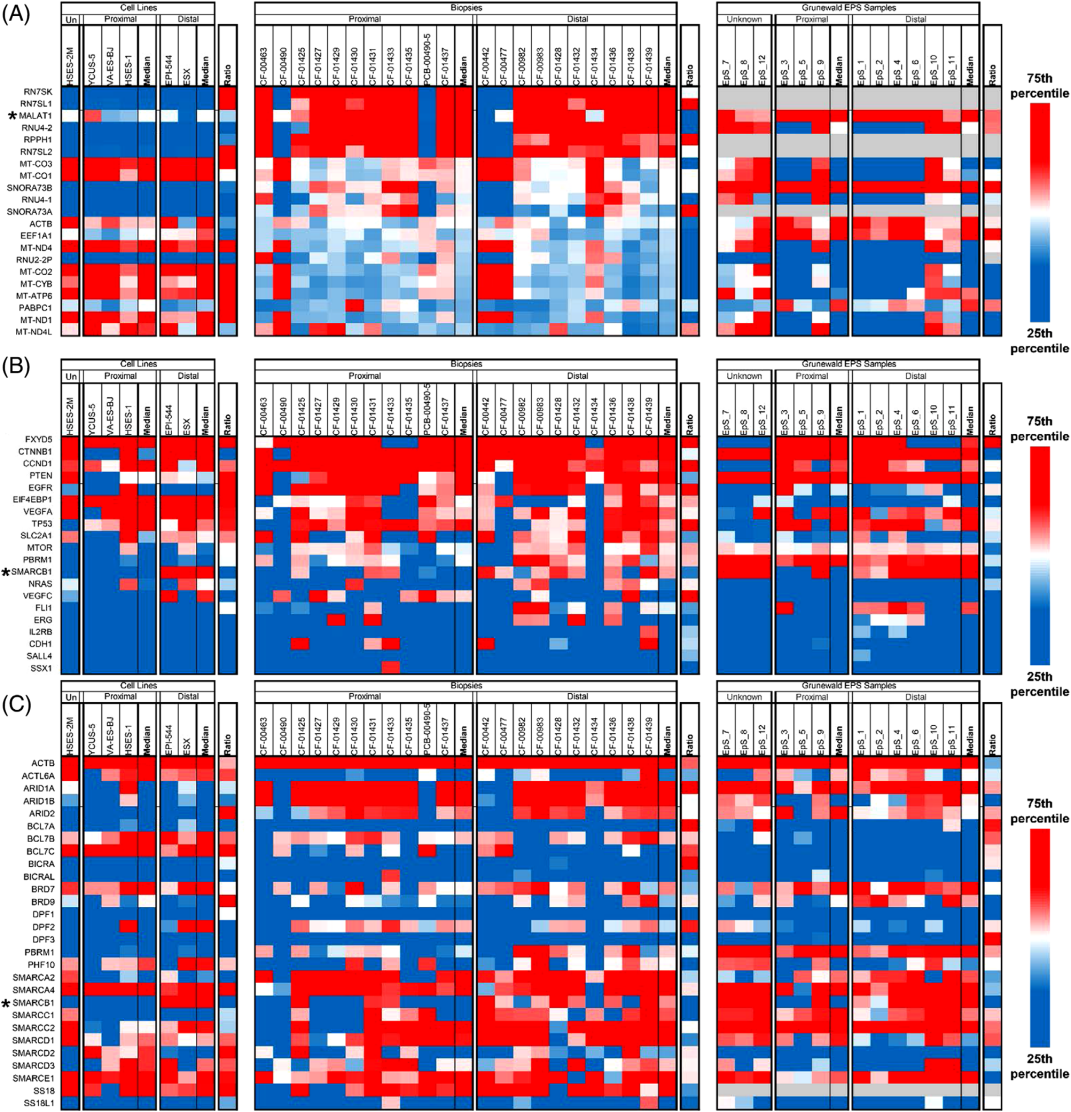
Disease‐wide expression of gene cohorts of interest. Median = median of associated samples, ratio = ratio between proximal and distal median of the associated samples. (A) Expression of genes with the highest median expression across EPS samples. The long non‐coding RNA *MALAT1* has consistently high expression in biopsy‐origin samples and lower expression in cell lines. (B) Expression of previously summarised potentially actionable biomarkers. Only *SMARCB1* expression significantly differs between distal and proximal samples. (C) Expression of the BAF, PBAC and ncBAF complex components. Only *SMARCB1* expression significantly differs between distal and proximal samples

Analysis of the RNA expression data from the EPS cohort identified the highest overall median expression occurred in RNA processing genes and mitochondrial genes (Figure 6A). None of the highest‐expressing genes demonstrated significantly different expression between proximal and distal biopsy cohorts from two independent laboratories (Keller Lab and Grünewald Lab), before or after correcting for multiple comparisons.

We also compared gene expression of twenty (20) genes previously summarised as potential EPS biomarkers (Figure 6B).^6^ Three of the twenty biomarker genes were upregulated in distal EPS samples (*SMARCB1*, 3.1× overexpression, *SALL4*, 2× overexpression and *FLI1*, 1.7× overexpression) and one gene upregulated in proximal EPS samples (*CCND1*, 1.1× overexpression). However, the significance identified did not hold true under multiple comparison testing.

*SMARCB1* expression was also significantly upregulated in distal cell lines versus proximal cell lines (*p* < .01) but significance did not hold true under multiple comparison testing. *SMARCB1* was also statistically upregulated in Keller Lab origin tissue samples (*p* < .05) and all Keller Lab origin distal samples versus proximal samples (*p* < .01). Notably, *SMARCB1* and in all distal samples (biopsy and cell line together) versus proximal samples (*p* < .001), which was significant even after multiple comparison testing. Consistent differential expression of *SMARCB1* coupled with the importance of *SMARCB1* to EPS aetiology suggests there may be fundamental differences between the two subtypes connected to, or beyond, site of origin.

We also analysed expression of the individual members of the *BAF*, *PBAC* and *ncBAF* due to critical roles in tumourigenesis of EPS (Figure 6C). Beyond *SMARCB1*, only *ACTL6A* showed significantly different expression (1.4× overexpression in proximal biopsy samples, *p* < .05), but the result was not significant after multiple comparison testing.

Given previous studies suggesting the importance of the extracellular matrix (ECM) in sarcoma,^17^ we investigated the expression patterns of extracellular matrix genes in EPS (Figure S6). Four ECM genes were significantly overexpressed in distal EPS (*COL4A1*, 3.3× overexpression, *COL5A*, 4.2× overexpression, *COL12A1*, 2.5× overexpression, *COLEC12*, 1.5× overexpression), but significance did not hold true under multiple comparison testing.

**FIGURE 7:**
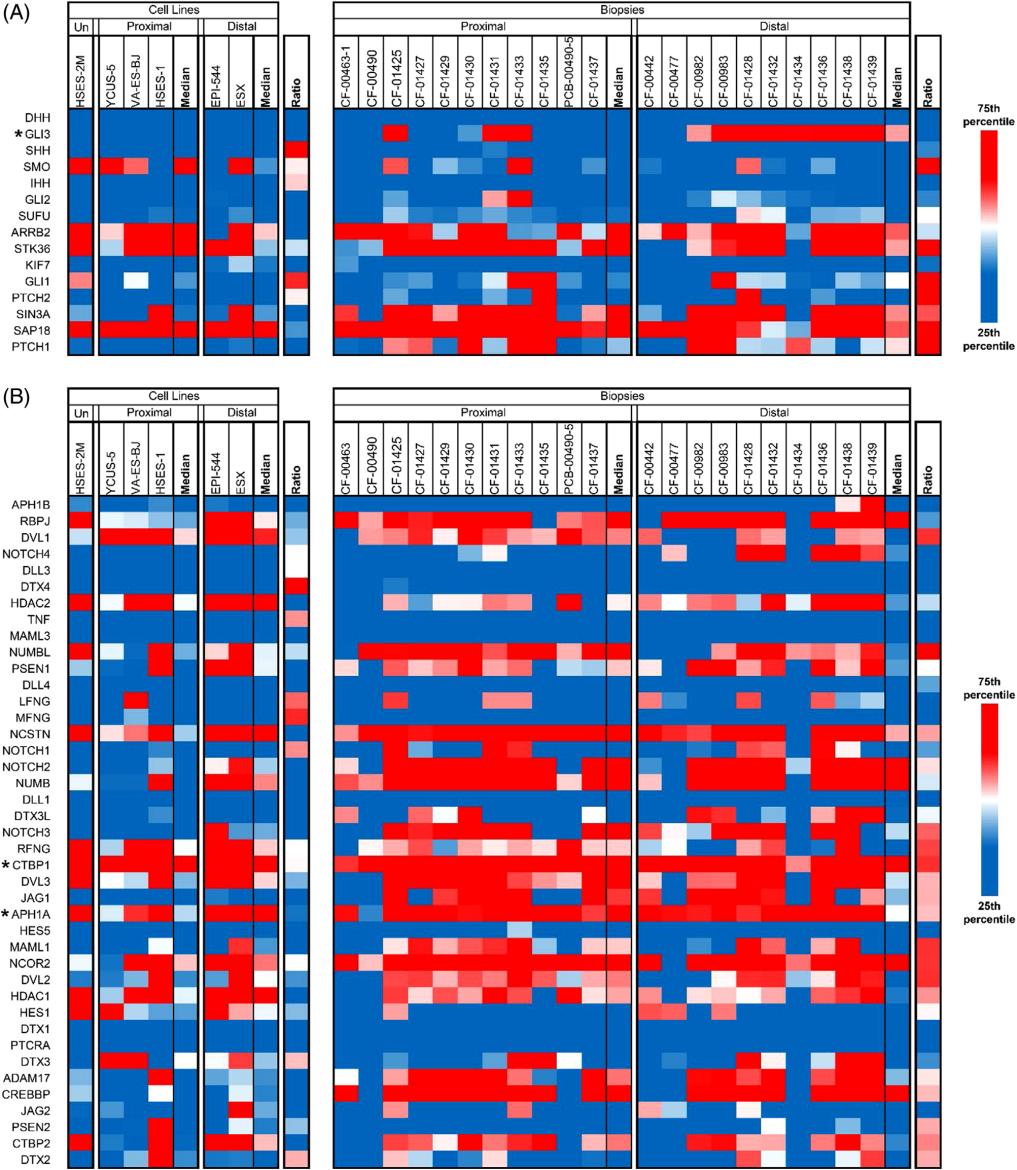
Expression of Sonic Hedgehog and notch pathway genes. (A) Expression of Sonic Hedgehog (*SHH*)‐associated genes. *GLI3* is over‐expressed in distal versus proximal biopsies, but not in cell lines. (B) Expression of Notch‐associated genes. *CTBP1* is consistently overexpressed and is the target of small molecule NSC95397. The gamma secretase subunit *APHA1A* is also consistently expressed in both subtypes. Nirogacestat, a clinically investigated gamma secretase inhibitor, may be a viable therapeutic.

**FIGURE 8:**
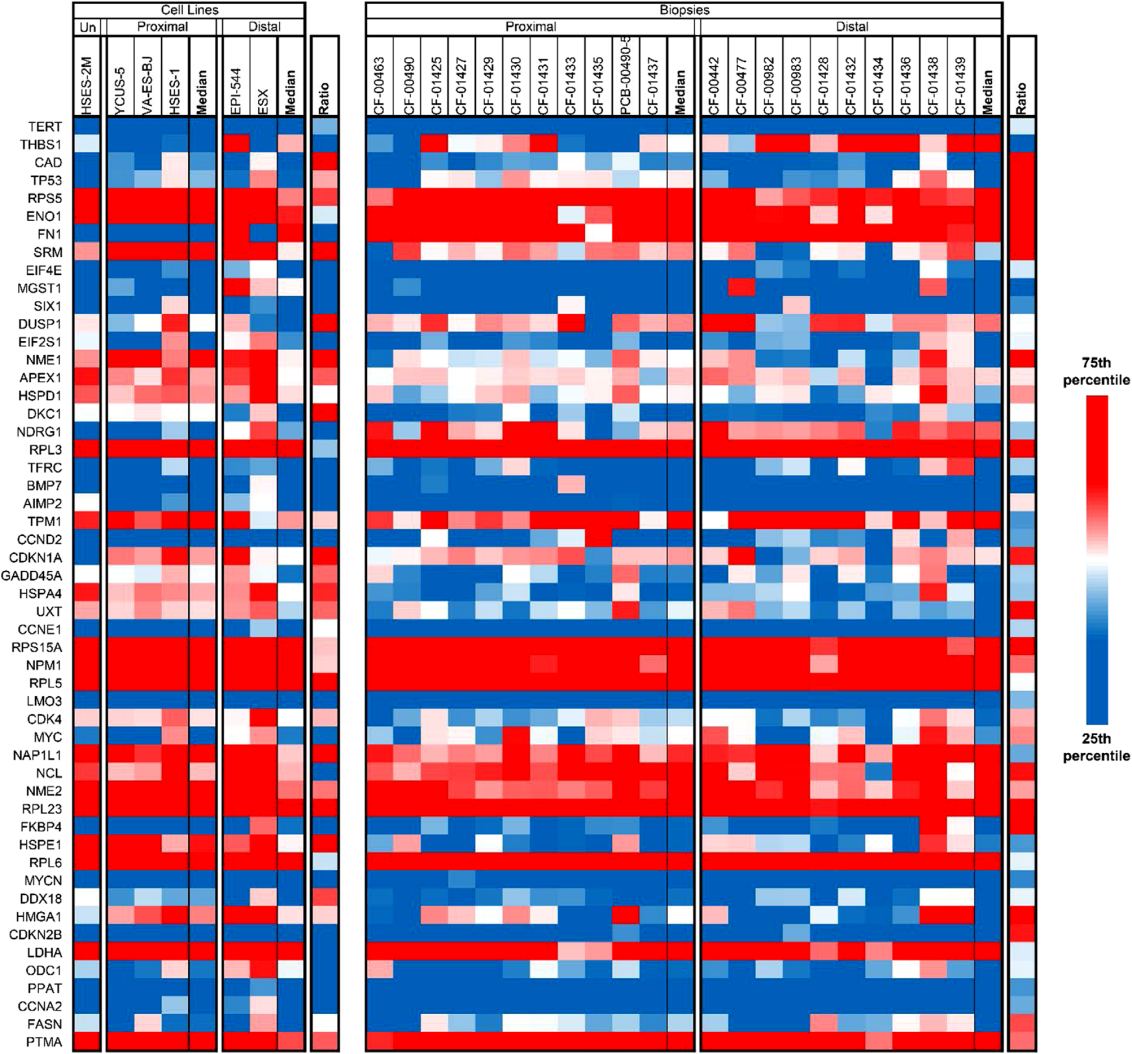
Expression of MYC pathways genes. *THBS1* (thrombospondin 1) is more highly expressed in distal than proximal biopsy samples; however, significance was not present adjusted for multiple comparison testing. All other MYC pathway genes were not significantly differentially expressed

Finally, a previous study had identified the MYC pathway as overexpressed in proximal EPS versus distal EPS, and the Sonic Hedgehog (*SHH*) and Notch pathways as overexpressed in distal EPS versus proximal EPS.^18^ In the *SHH* pathway, only *DHH* 3.2× overexpression) was differentially expressed in distal versus proximal EPS after multiple comparison correction (Figure 7A). In the Notch and MYC pathways, no genes were differentially expressed in distal versus proximal EPS after multiple comparison testing (Figures 7B and 8). Overall, the three previously identified signalling pathways were not significantly differentially expressed within the current cohort of EPS samples.

All statistical analyses are presented in Table S3. The complete set of DNA whole exome and RNA whole transcriptome sequencing data are provided as supplementary data.

### 3.6 Targeting PABPC1 in EPS

As stated earlier, gene expression analysis also identified *PABPC1* as a highly expressed target in EPS, as are four additional *PABP*‐family genes: *PABPN1*, *PABPC4*, *PABPC1L* and *PABPC1P3* (Figure S7A). PABPC1 promotes ribosome recruitment/translation and is critical in the first step of mRNA decay.^19^ Correspondingly, we investigated pharmacological effect of *PABPC1* siRNA knockdown in combination with autophagy inhibitor hydroxychloroquine (HCQ) or nuclear export inhibitor selinexor (Figure S7B and C). We confirmed *PABPC1* knockdown following siRNA exposure via protein quantification (Figure S7D). While both agents induced changes in cell growth in a concentration‐dependent manner for EPS cell line VA‐ES‐BJ, drug effect was independent of *PABPC1* siRNA knockdown suggesting that PABPC1 targeting in conjunction with autophagy or nuclear export inhibition may not be an effective therapeutic strategy for EPS.

### 3.7 Therapeutic compound screen and validation studies on EPS cell models

Having confirmed *SMARCB1* status of EPS cell models, we proceeded to screen eight EPS cell models, two *SMARCB1*‐null rhabdoid cell models and two normal cell lines against a custom compound library (Figure S8) consisting of 61 pre‐clinical and clinical agents selected based on relevance to biological pathways implicated in sarcoma and targets currently under investigation as therapeutic mechanisms in sarcoma (Table 3). Overall results from the compound screen measuring 72‐h cell viability demonstrated a lack of response specificity to either EPS cell models versus non‐EPS cell models, and a lack of specificity to *SMARCB1*‐null cell models versus *SMARCB1–*expressing cell models (Figure S8). Nonetheless, we identified a subset of non‐differentially active agents and pursued further validation experiments (Figure S9A–D) using a set of PI3K pathway inhibitors (BEZ235, XL765, INK128, BKM120 and BYL719) and multi‐kinase inhibitors (dasatinib, sunitinib and sorafenib). The follow‐on validation studies did not demonstrate consistent sensitivity for a single agent across all tested cell models, although dasatinib (multi‐kinase inhibitor) and BEZ235 (PI3K/mTOR inhibitor) performed comparatively better than other pathway inhibitors. Correspondingly, we performed a checkerboard concentration study to determine synergy between BEZ235 and dasatinib, which demonstrated synergy at clinically relevant concentrations (dasatinib *C*_max_ ≈ 160 nM, BEZ235 *C*_max_ ≈ 457 nM)^20^, ^21^ (Figure S9E)

### 3.8 Tazemetostat‐focused combination studies in EPS cell models

Following the lack of single agents active at clinically feasible concentrations for the high‐throughput in vitro chemical screen, and given the recent clinical trials demonstrating tazemetostat efficacy, we investigated combinations built around tazemetostat pre‐treatment as potential EPS treatments. Given previously published synergy between EZH2 inhibitors (tazemetostat) and bromodomain inhibitors (BRDi),^22^ we investigated the effect of tazemetostat pre‐treatment on response of bromodomain inhibitors (+)‐JQ1 and UNC0642 (Figure S10). While pre‐treatment generally decreases in vitro BRDi IC_50_ concentrations, tazemetostat pre‐treatment does not significantly alter the response of either BRDi agent.

The lack efficacy of BRDi small molecule inhibitor agents led us to confirm the biological effect of the BRD7/9 PROTACs VZ185 and cis‐VZ185 (Figure S11). Functional response of PROTAC monotherapy demonstrates response only at high concentrations, suggesting the monotherapy PROTAC is unlikely to be an effective therapy for EPS. Similarly, the combination of tazemetostat with phosphodiesterase agents modulating oxidative phosphorylation had no demonstrable effect on EPS tumour cell growth (Figure S12).

## 4 DISCUSSION

Herein we report the most comprehensive study to date of the functional genomic landscape of EPS. Across the largest cohort of EPS cell line models and patient‐origin biopsy tissues to date, no consistent secondary mutation was found. DNA variations and RNA expression showed PABPC1 as a potential target, but in our interference experiment, we did not observe an effect on autonomous cell viability. Additional functional validations were equally non‐informative, underlining the complexity of EPS and the need for deeper and more wide‐ranging functional studies in a larger chemical space.

A key finding in this study was the identification of biological differences between distal EPS (common in children and young adults, often having a favourable diagnosis) and proximal EPS (more common in adults). Specifically, molecular sequencing and immunocytochemical staining of *SMARCB1* generally demonstrates deletion in proximal EPS samples, while distal EPS samples show a pattern of retained and/or presumably dysfunctional *SMARCB1*. While distal and proximal EPS generally demonstrate similar gene expression patterns across the broader transcriptome, the fundamental biological difference observed in the pathogenic initiator of EPS corresponds with differential expression patterns in genes associated with and connected to *SMARCB1*. Differentially expressed genes include directly or indirectly actionable molecular targets, such as *FYN, GLI3* and *CXCL12*. Retained expression in distal EPS enables therapeutic targeting of *SMARCB1* through protein degradation platforms. While traditionally viewed as a tumour suppressor gene in the context of rhabdoid tumours and EPS, targeting of retained *SMARCB1* merits functional investigation.

## 5 CONCLUSION

Overall results of our functional genomic investigation of EPS highlight the complexity of the disease and the current limited knowledge of the optimal chemical space for therapeutic advancement. Nonetheless, the subtle differences in the initiating pathogenic alteration between the two EPS subtypes highlights the biological disparities between younger and older EPS patients and emphasises the need to approach the two subtypes as molecularly and clinically distinct diseases. Long non‐coding RNA's and miRNA's may be an area of further exploration.

## CONFLICT OF INTEREST

The authors declare no direct conflicts of interest with respect to these studies. CK has sponsored research agreements with Eli Lily, Roche‐Genentech and Cardiff Oncology as well as research collaborations with Novartis, and is co‐founder of Tio Companies. Artisan Biopharma is a wholly owned subsidiary of cc‐TDI.

## Supporting information



Supplementary information

## References

[1] Jawad MU , Extein J , Min ES , Scully SP . Prognostic factors for survival in patients with epithelioid sarcoma: 441 cases from the SEER database. Clin Orthop Relat Res. Nov 2009;467(11):2939‐2948. 10.1007/s11999-009-0749-2 19224301 PMC2758965

[2] Jones RL , Constantinidou A , Olmos D , et al. Role of palliative chemotherapy in advanced epithelioid sarcoma. Am J Clin Oncol. Aug 2012;35(4):351‐357. 10.1097/COC.0b013e3182118cf7 21422990

[3] Enzinger FM . Epitheloid sarcoma. A sarcoma simulating a granuloma or a carcinoma. Cancer. Nov 1970;26(5):1029‐1041. 10.1002/1097-0142(197011)26:5<1029::aid‐cncr2820260510>3.0.co;2‐r 5476785

[4] Chbani L , Guillou L , Terrier P , et al. Epithelioid sarcoma: a clinicopathologic and immunohistochemical analysis of 106 cases from the French sarcoma group. Am J Clin Pathol. Feb 2009;131(2):222‐227. 10.1309/ajcpu98abipvjaiv 19141382

[5] Chase DR , Enzinger FM . Epithelioid sarcoma. Diagnosis, prognostic indicators, and treatment. Am J Surg Pathol. Apr 1985;9(4):241‐263. 4014539

[6] Noujaim J , Thway K , Bajwa Z , et al. Epithelioid sarcoma: opportunities for biology‐driven targeted therapy. Front Oncol. 2015;5:186. 10.3389/fonc.2015.00186 26347853 PMC4538302

[7] Jamshidi F , Bashashati A , Shumansky K , et al. The genomic landscape of epithelioid sarcoma cell lines and tumours. J Pathol. Jan 2016;238(1):63‐73. 10.1002/path.4636 26365879

[8] Berlow NE , Rikhi R , Geltzeiler M , et al. Probabilistic modeling of personalized drug combinations from integrated chemical screen and molecular data in sarcoma. BMC Cancer. Jun 17 2019;19(1):593. 10.1186/s12885-019-5681-6 31208434 PMC6580486

[9] Zaffaroni N , Frezza AM , Zuco V , et al. Doxorubicin (D), gemcitabine (G), ifosfamide (I) and the EZH2 inhibitor EPZ‐011989 in epithelioid sarcoma (ES): a comparison of different regimens in a patient‐derived xenograft (PDX) model. J Clin Oncol. 2018;36(15_suppl):11578. 10.1200/JCO.2018.36.15_suppl.11578

[10] Gounder M , Schöffski P , Jones RL , et al. Tazemetostat in advanced epithelioid sarcoma with loss of INI1/SMARCB1: an international, open‐label, phase 2 basket study. Lancet Oncol. Nov 2020;21(11):1423. 10.1016/s1470-2045(20)30451-4 33035459

[11] Sullivan LM , Folpe AL , Pawel BR , Judkins AR , Biegel JA . Epithelioid sarcoma is associated with a high percentage of SMARCB1 deletions. Mod Pathol. Mar 2013;26(3):385‐392. 10.1038/modpathol.2012.175 23060122 PMC3556344

[12] Nakayama RT , Pulice JL , Valencia AM , et al. SMARCB1 is required for widespread BAF complex‐mediated activation of enhancers and bivalent promoters. Nat Genet. Nov 2017;49(11):1613‐1623. 10.1038/ng.3958 28945250 PMC5803080

[13] Isakoff MS , Sansam CG , Tamayo P , et al. Inactivation of the Snf5 tumor suppressor stimulates cell cycle progression and cooperates with p53 loss in oncogenic transformation. Proc Natl Acad Sci U S A. Dec 6 2005;102(49):17745‐17750. 10.1073/pnas.0509014102 16301525 PMC1308926

[14] Lupo A , Cesaro E , Montano G , Zurlo D , Izzo P , Costanzo P . KRAB‐zinc finger proteins: a repressor family displaying multiple biological functions. Curr Genomics. 2013;14(4):268‐278. 10.2174/13892029113149990002 24294107 PMC3731817

[15] Urakawa H , Kawai A , Goto T , et al. Phase II trial of pazopanib in patients with metastatic or unresectable chemoresistant sarcomas: a Japanese Musculoskeletal Oncology Group study. Cancer Sci. 2020;111(9):3303‐3312. 10.1111/cas.14542 32579783 PMC7469808

[16] Irimura S , Nishimoto K , Kikuta K , et al. Successful treatment with pazopanib for multiple lung metastases of inguinal epithelioid sarcoma: a case report. Case Rep Oncol. 2015;8(3):378‐384. 10.1159/000439427 26500539 PMC4608657

[17] Lian X , Bond JS , Bharathy N , et al. Defining the Extracellular matrix of rhabdomyosarcoma. Front Oncol. 2021;11:601957. 10.3389/fonc.2021.601957 33708626 PMC7942227

[18] Czarnecka AM , Sobczuk P , Kostrzanowski M , et al. Epithelioid sarcoma‐from genetics to clinical practice. Cancers (Basel). 2020;12(8) 10.3390/cancers12082112 PMC7463637 32751241

[19] Mattijssen S , Kozlov G , Fonseca BD , Gehring K , Maraia RJ . LARP1 and LARP4: up close with PABP for mRNA 3' poly(A) protection and stabilization. RNA Biol. 2021;18(2):259‐274. 10.1080/15476286.2020.1868753 33522422 PMC7928012

[20] Demetri GD , Lo Russo P , MacPherson IR , et al. Phase I dose‐escalation and pharmacokinetic study of dasatinib in patients with advanced solid tumors. Clin Cancer Res. Oct 1 2009;15(19):6232‐6240. 10.1158/1078-0432.Ccr-09-0224 19789325

[21] Wise‐Draper TM , Moorthy G , Salkeni MA , et al. A Phase Ib study of the dual PI3K/mTOR inhibitor dactolisib (BEZ235) combined with everolimus in patients with advanced solid malignancies. Target Oncol. 2017;12(3):323‐332. 10.1007/s11523-017-0482-9 28357727 PMC5447332

[22] Shorstova T , Foulkes WD , Witcher M . Achieving clinical success with BET inhibitors as anti‐cancer agents. Br J Cancer. 2021;124(9):1478‐1490. 10.1038/s41416-021-01321-0 33723398 PMC8076232

[23] Emori M , Tsukahara T , Murase M , et al. High expression of CD109 antigen regulates the phenotype of cancer stem‐like cells/cancer‐initiating cells in the novel epithelioid sarcoma cell line ESX and is related to poor prognosis of soft tissue sarcoma. PLoS One. 2013;8(12):e84187. 10.1371/journal.pone.0084187 24376795 PMC3869840

[24] Goto H , Takahashi H , Funabiki T , Ikuta K , Sasaki H , Nagashima Y . Brief report: neural differentiation of a novel cell line, YCUS‐5, established from proximal‐type epithelioid sarcoma of a child. Med Pediatr Oncol. 1999;33(2):137‐138. 10.1002/(sici)1096-911x(199908)33:2<137::aid‐mpo18>3.0.co;2‐n 10398195

[25] Zhang B , Horvath S . A general framework for weighted gene co‐expression network analysis. Stat Appl Genet Mol Biol. 2005;4:Article17. 10.2202/1544-6115.1128 16646834

[26] Langfelder P , Horvath S . WGCNA: an R package for weighted correlation network analysis. BMC Bioinfo. 2008;9:559. 10.1186/1471-2105-9-559 PMC2631488 19114008

[27] Huang da W , Sherman BT , Lempicki RA . Bioinformatics enrichment tools: paths toward the comprehensive functional analysis of large gene lists. Nucleic Acids Res. 2009;37(1):1‐13. 10.1093/nar/gkn923 19033363 PMC2615629

[28] Benjamini Y , Hochberg Y . Controlling the false discovery rate: a practical and powerful approach to multiple testing. J R Stat Soc: Ser B (Methodol). 1995;57(1):289‐300. 10.1111/j.2517-6161.1995.tb02031.x

[29] Berlow NE . Probabilistic Boolean modeling of pre‐clinical tumor models for biomarker identification in cancer drug development. Curr Protoc. 2021;1(10):e269. 10.1002/cpz1.269 34661991

[30] Bacher JW , Flanagan LA , Smalley RL , et al. Development of a fluorescent multiplex assay for detection of MSI‐High tumors. Dis Markers. 2004;20(4‐5):237‐250. 10.1155/2004/136734 15528789 PMC3839403

[31] Niu B , Ye K , Zhang Q , et al. MSIsensor: microsatellite instability detection using paired tumor‐normal sequence data. Bioinformatics. 2014;30(7):1015‐1016. 10.1093/bioinformatics/btt755 24371154 PMC3967115

[32] Sonobe H , Ohtsuki Y , Sugimoto T , Shimizu K . Involvement of 8q, 22q, and monosomy 21 in an epithelioid sarcoma. Cancer Genet Cytogenet. 1997;96(2):178‐180. 10.1016/s0165‐4608(96)00412‐8 9216728

[33] Helson C , Melamed M , Braverman S , Traganos F , Preti R , Helson L . Va‐es‐bj – an epithelioid sarcoma cell‐line. Int J Oncol. 1995;7(1):51‐56. 10.3892/ijo.7.1.51 21552805

[34] Sakharpe A , Lahat G , Gulamhusein T , et al. Epithelioid sarcoma and unclassified sarcoma with epithelioid features: clinicopathological variables, molecular markers, and a new experimental model. Oncologist. 2011;16(4):512‐522. 10.1634/theoncologist.2010-0174 21357725 PMC3228127

