# Supplementary material for: Functional genomic analysis of epithelioid sarcoma reveals distinct proximal and distal subtype biology
Source: Clin Transl Med. 2022 Jul 15;12(7):e961. doi: 10.1002/ctm2.961 (PMC9286527; doi:10.1002/ctm2.961)
Supplement: Supplementary file 1 — Supplementary information [file CTM2-12-e961-s001.docx]

# Supplementary Figures


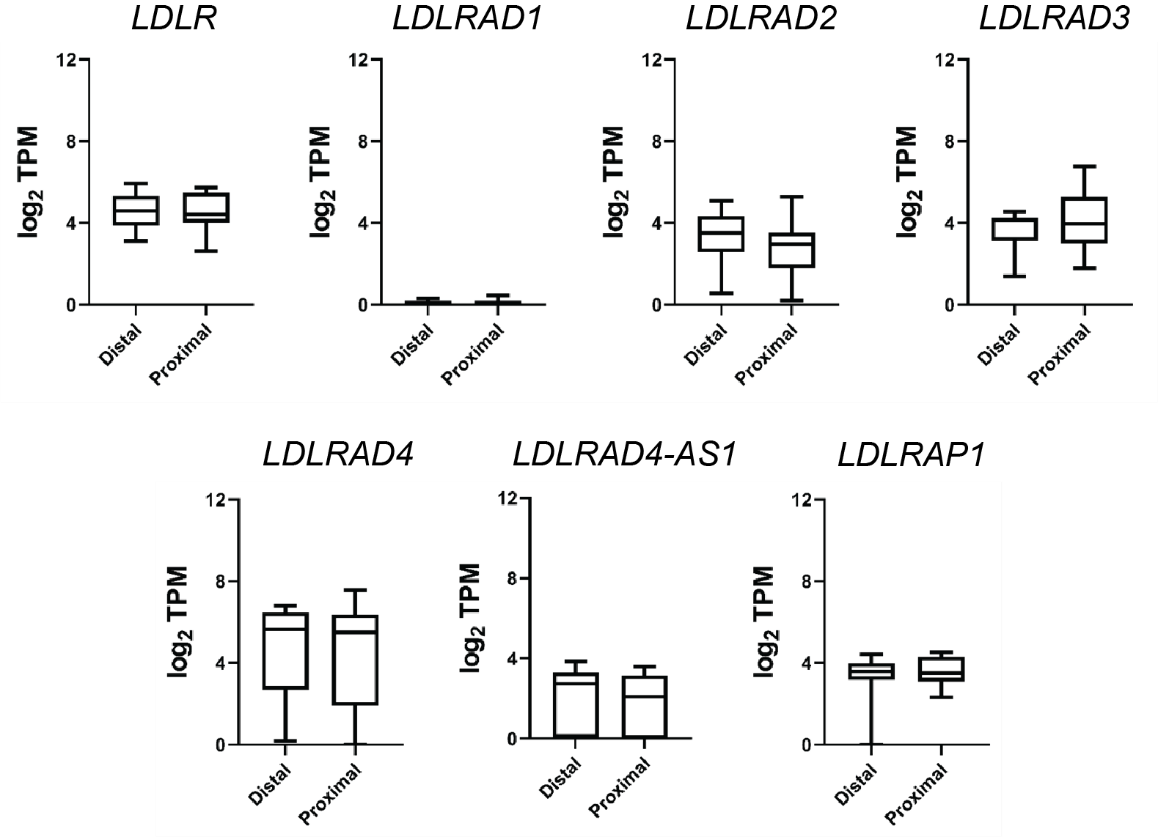

**Supplementary Fig 1. Expression distributions of LDL family genes.** Gene expression distributions for LDL family genes found to be enriched through eigengene analysis. No statistical significance was found through Mann-Whitney U-test analysis.

*
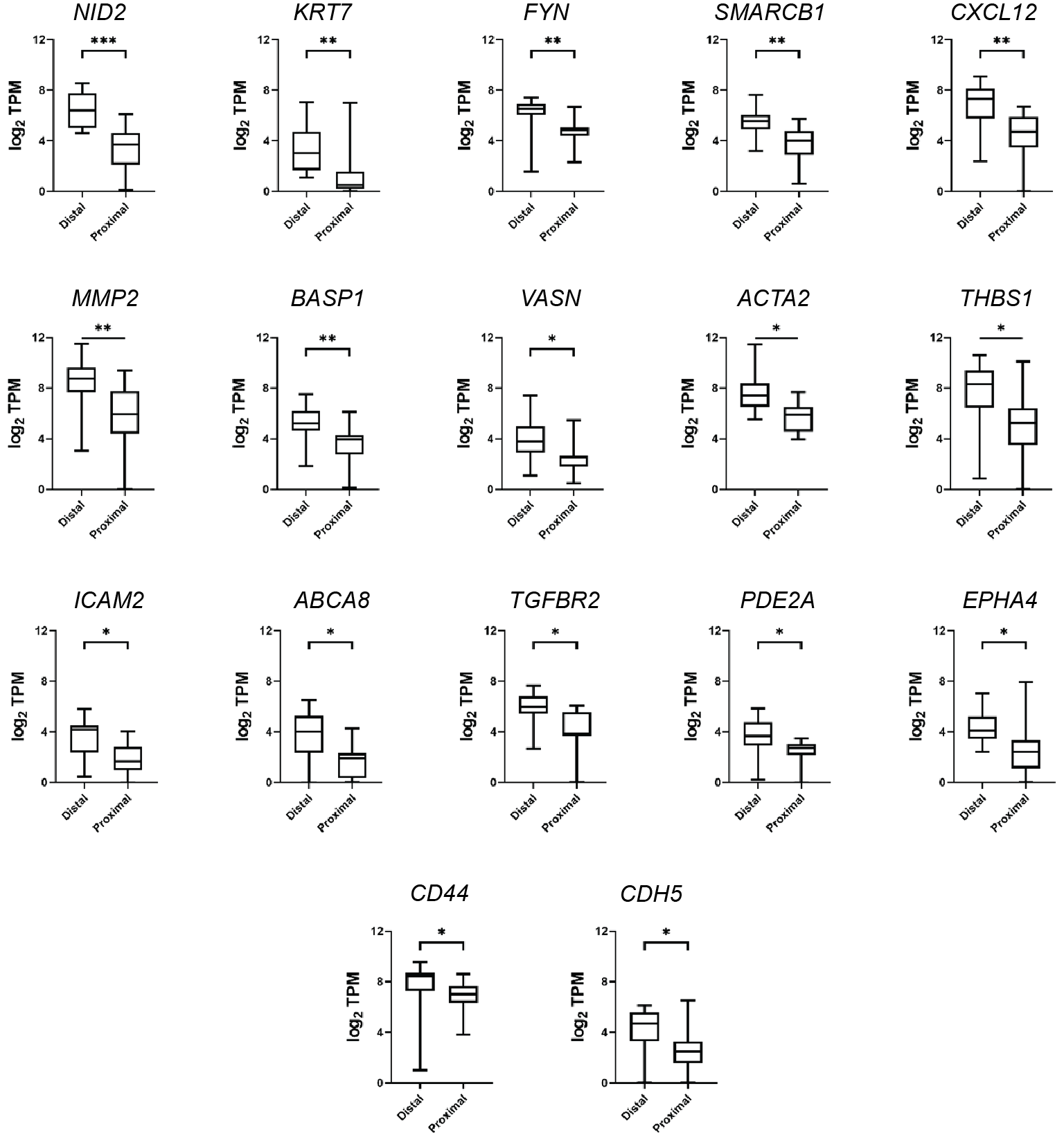
*

**Supplementary Fig 2. Expression distributions of genes significantly upregulated in distal EPS.** All gene expression distributions shown are significantly upregulated by Mann-Whitney U-test. *** represents p < 0.001, ** represents p < 0.01, * represents p < 0.05.


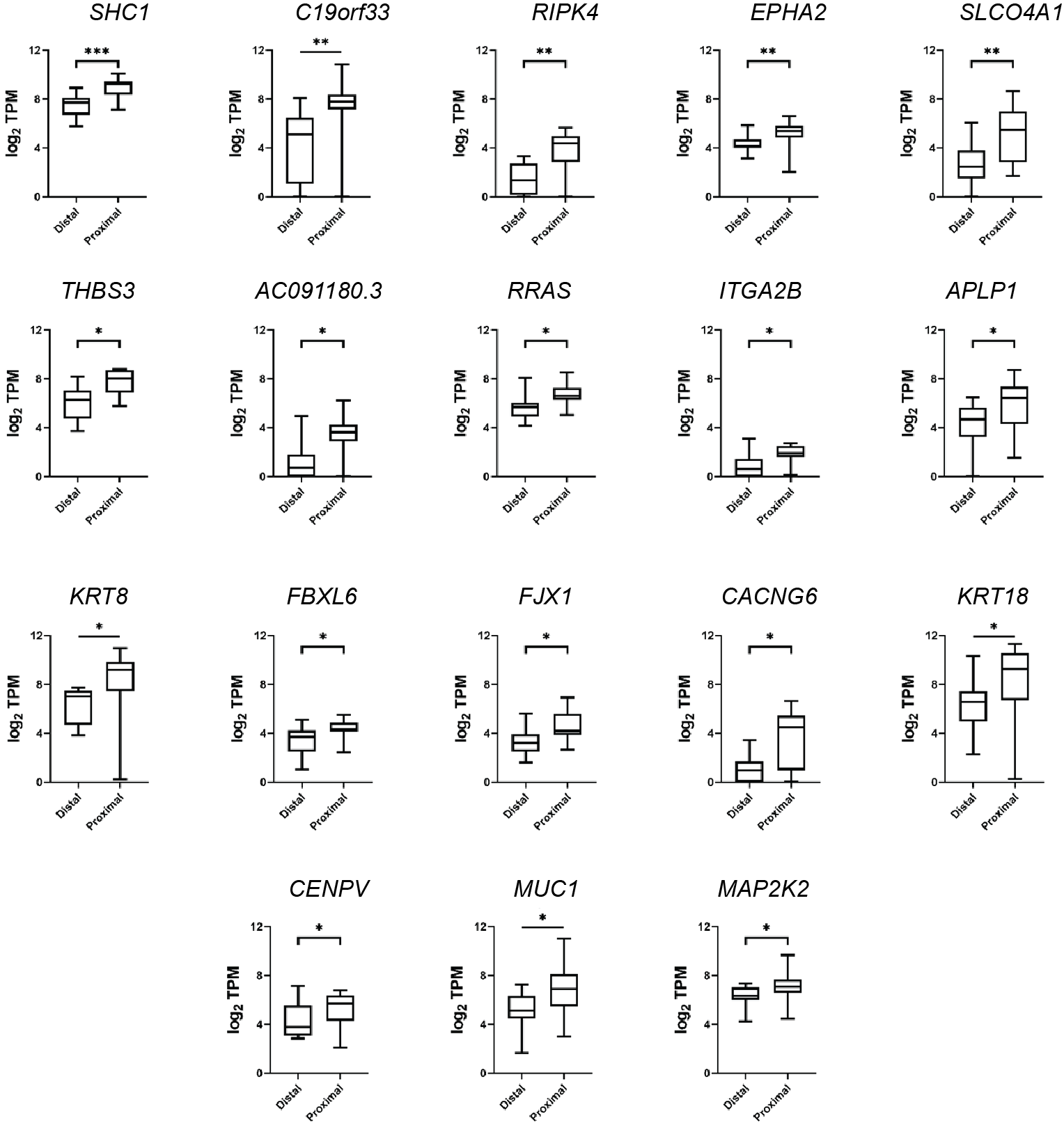


**Supplementary Fig 3. Expression distributions of genes significantly upregulated in proximal EPS. Expression distributions of genes upregulated in distal EPS.** All gene expression distributions shown are significantly upregulated by Mann-Whitney U-test. *** represents p < 0.001, ** represents p < 0.01, * represents p < 0.05.


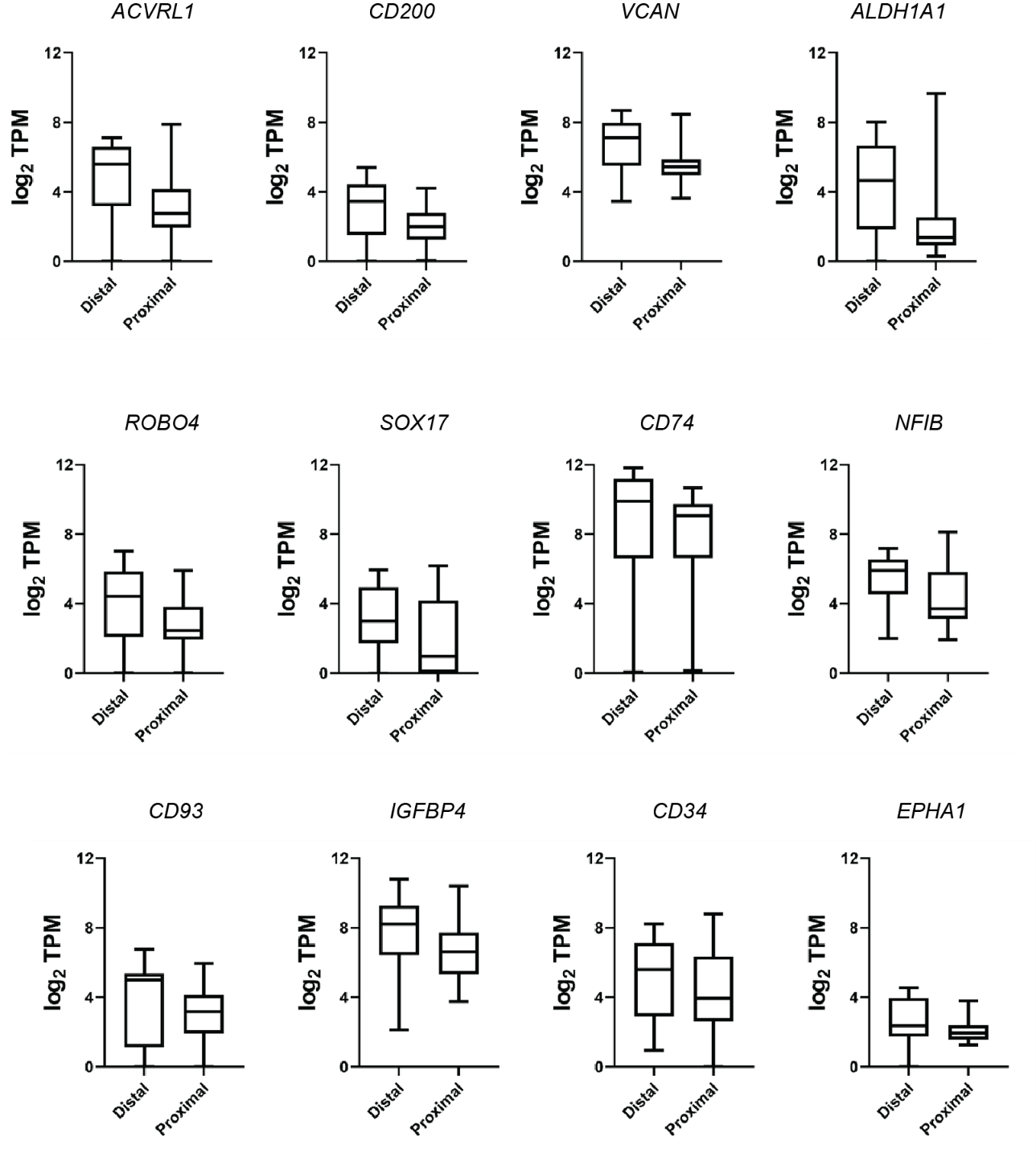


**Supplementary Fig 4. Expression distributions of genes upregulated in distal EPS.** Gene expression distributions shown are those identified by supervised learning proximal versus distal analysis and are not upregulated by Mann-Whitney U-test.


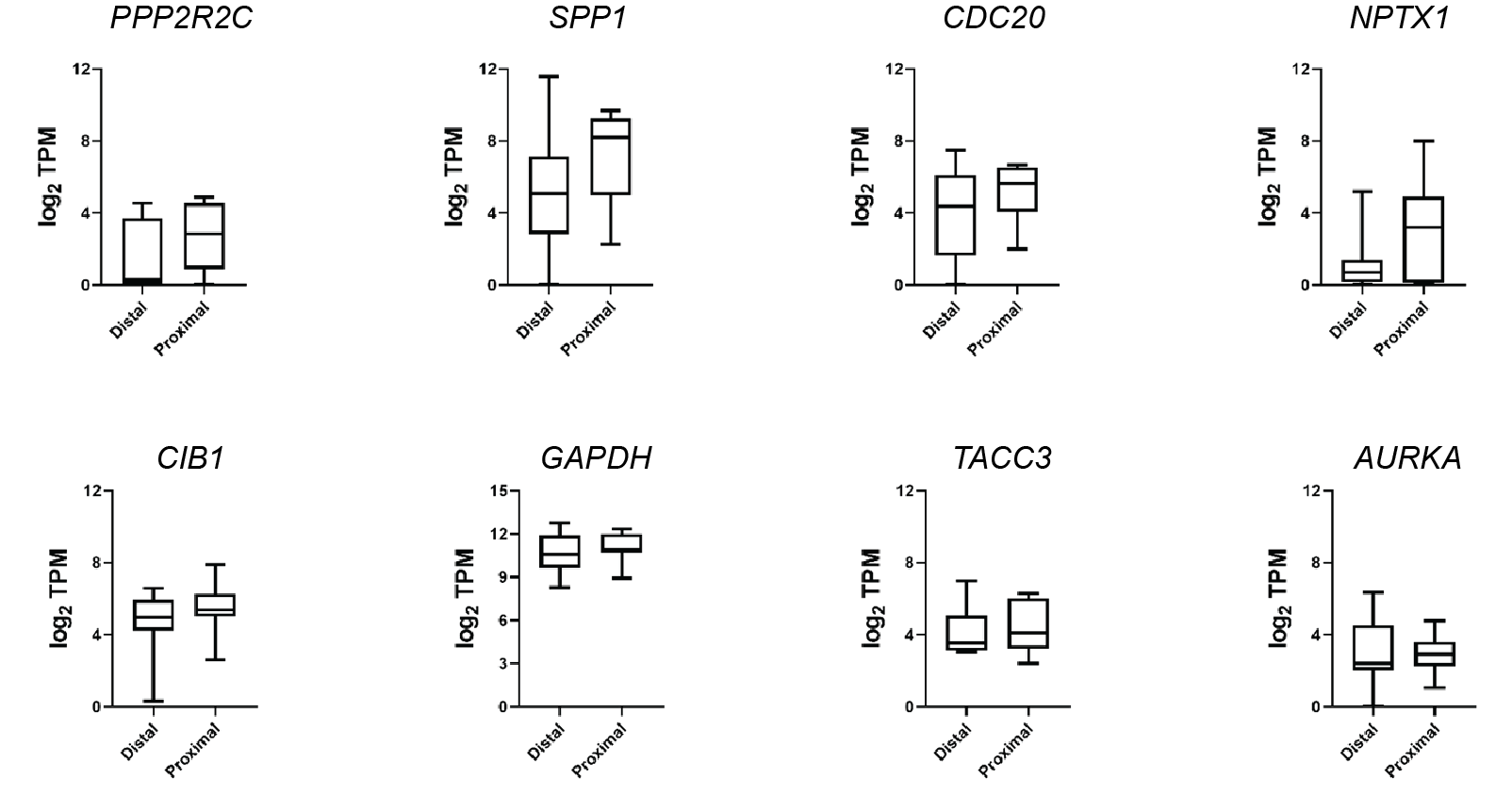

**Supplementary Fig 5. Expression distributions of genes upregulated in proximal EPS.** Gene expression distributions shown are those identified by supervised learning proximal versus distal analysis and are not upregulated by Mann-Whitney U-test.


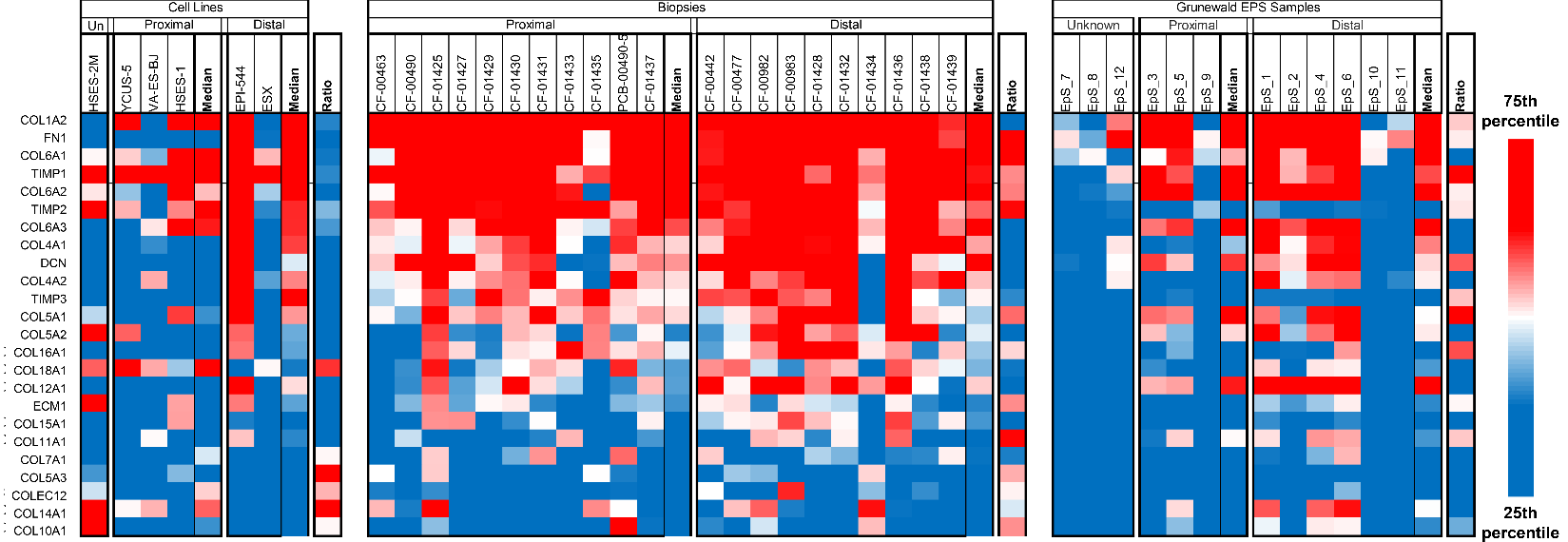


**Supplementary Fig** **6. RNAseq expression of ECM-associated genes.** Median = Median of associated samples, Ratio = Ratio between Proximal and Distal Median of the associated samples.


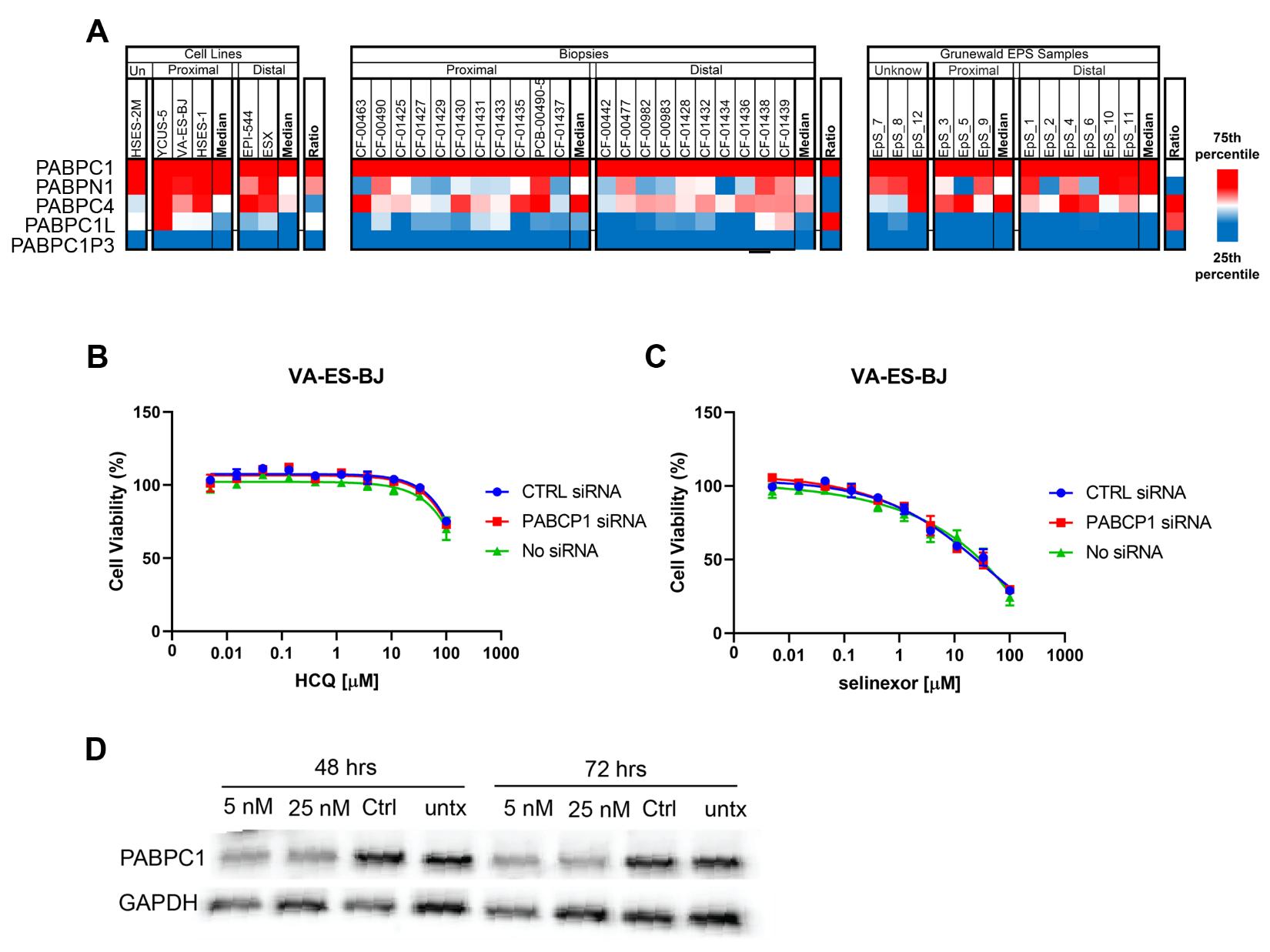


**Supplementary Fig 7. RNA interference of PABPC1 in EPS cell lines with and without autophagy inhibitors, with and without selinexor**. Median = Median of associated samples, Ratio = Ratio between Proximal and Distal Median of the associated samples. **a** Gene expression of PABPC1-associated genes. **b** Drug response curve of VA-ES-BJ cell line following PABCP1 siRNA knockdown and HCQ dosing. **c** Drug response curve of VA-ES-BJ cell line following PABCP1 siRNA knockdown and selinexor dosing. **d** Quantification of PABPC1 protein expression following PABCP1 siRNA knockdown.

**
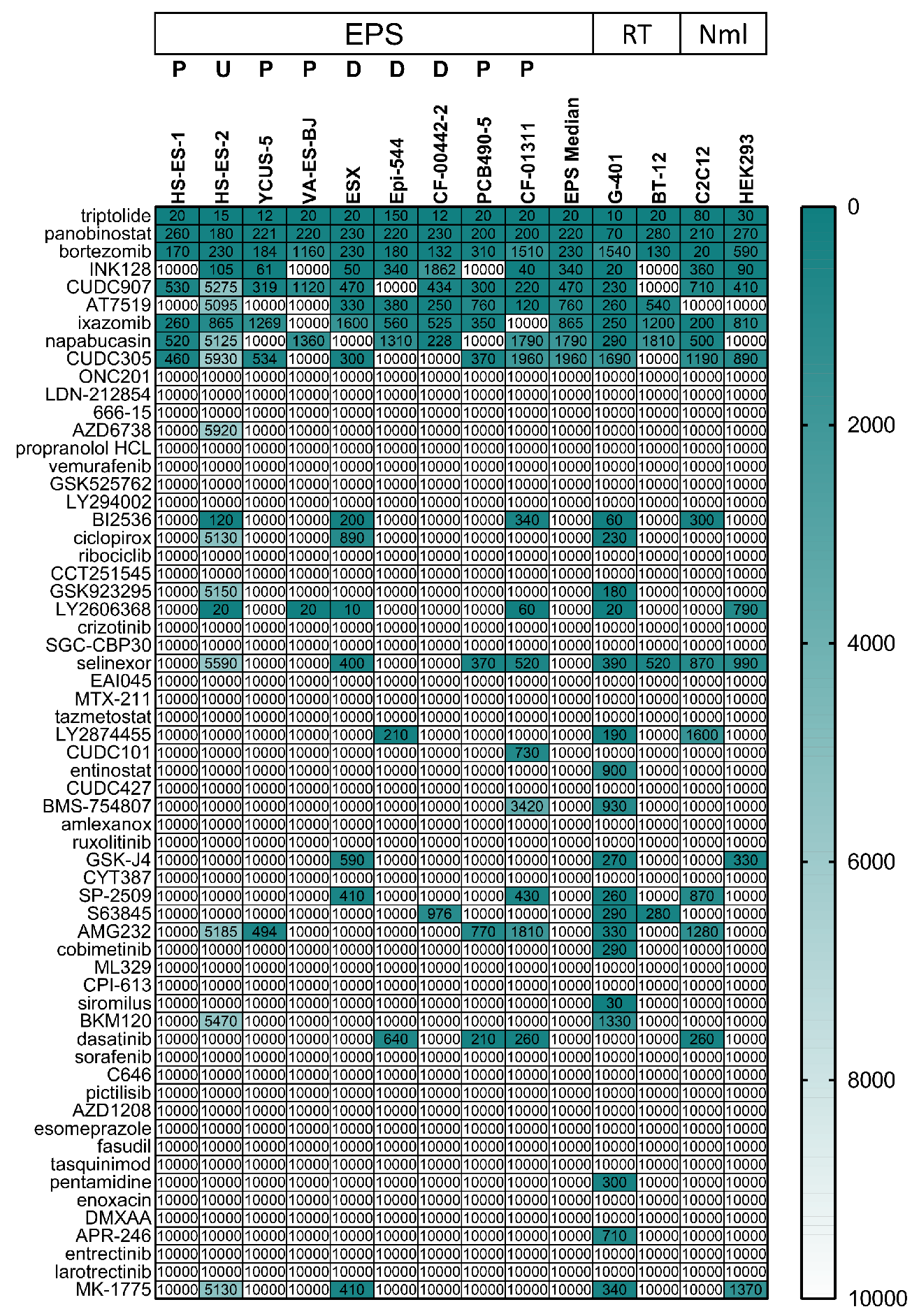
**

**Supplementary Fig 8. Focused single agent chemical screen of epithelioid sarcoma cell lines and controls.**  Absolute IC_50_ values (nM) for each cell line after 72-hour incubation with corresponding agent. A value of 10,000 indicates a lack of cell growth inhibition. Darker colors represent higher sensitivity. EPS = epithelioid sarcoma; RT = rhabdoid tumor; Nml = normal non-neoplastic cell culture. (Note: HS-ES-2 represents the median value of two different cell lines from the same patient, HS-ES-2M and HS-ES-2R). P = Proximal, D = Distal, U = Unknown. Location is anatomical, not histological.

**
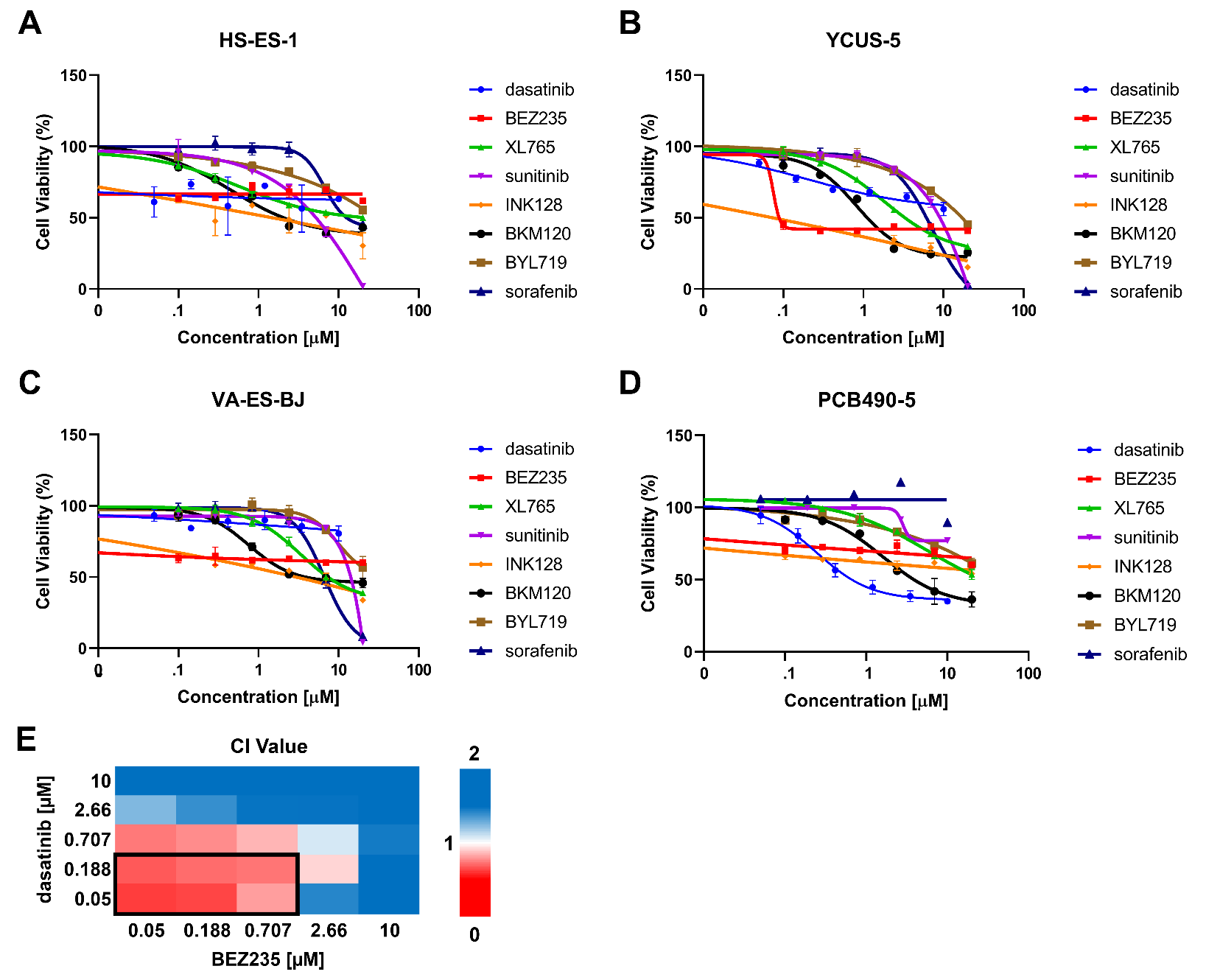
**

**Supplementary Fig 9. a** Monotherapy drug response in HE-ES-1 EPS cell line. **b** Monotherapy drug response in YCUS-5 EPS cell line. **c** Monotherapy drug response in VA-ES-BJ EPS cell line. **d** Monotherapy drug response in PCB490-5 EPS cell culture. **e** The Combination Index (synergy) of Pl3Ki BEZ235 and RTKi dasatinib. The dark border indicates the clinically-relevant dose combinations for the dasatinib and BEZ235.


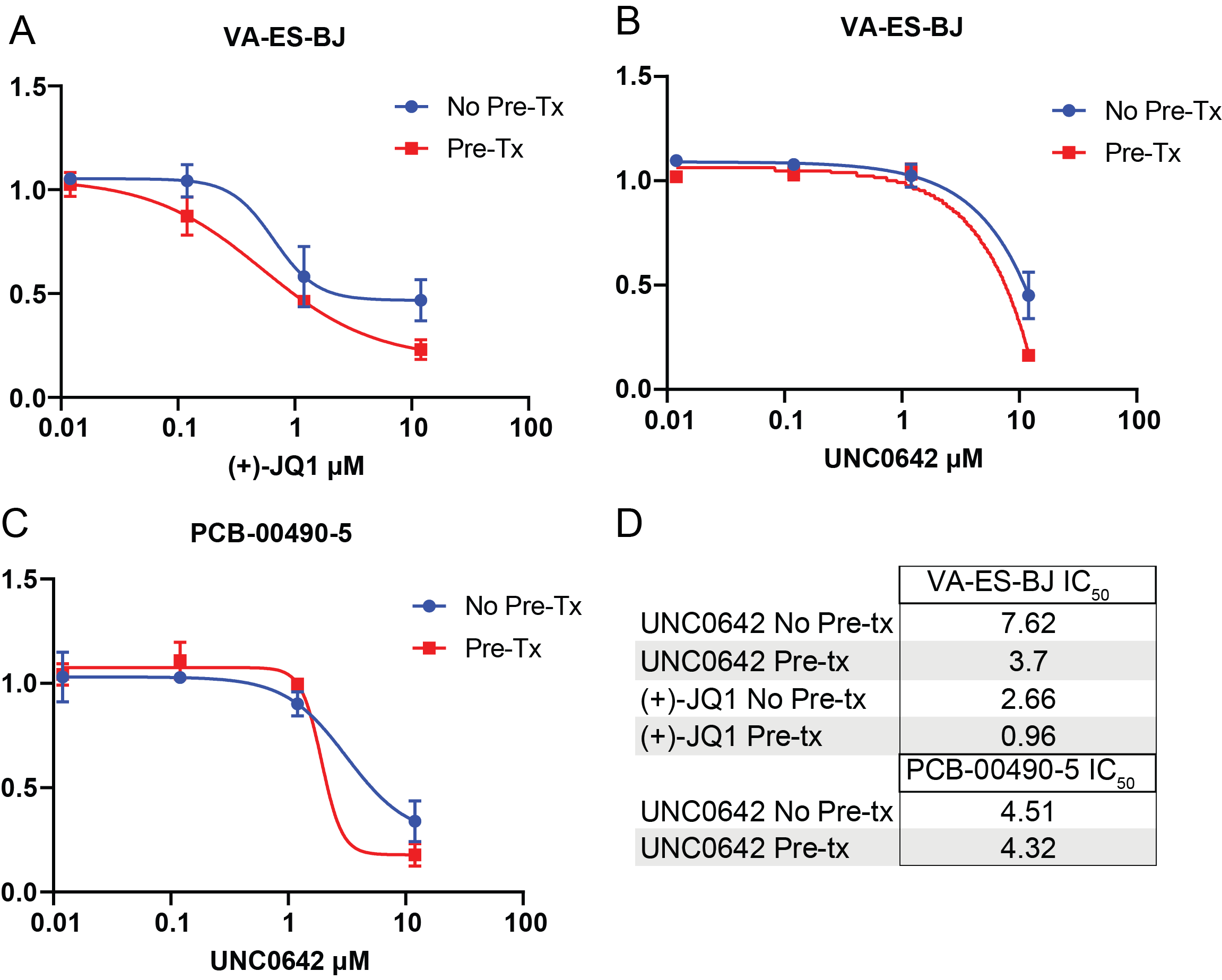


**Supplementary Fig 10. Epigenetic combination therapies in EPS cell lines using the SGC Library and tazemetostat (EZH2 inhibitor).** The pre-treatment is tazemetostat at 300 nM. **a-b** Inhibition of VA-ES-BJ using UNC0642 or (+)-JQ1 with and without pre-treatment. **c** inhibition of PCB-00490-5 using UNC0642 with and without pre-treatment. **d** relative IC_50s_ (which correspond to the dotted lines) and absolute IC_50s_.

**
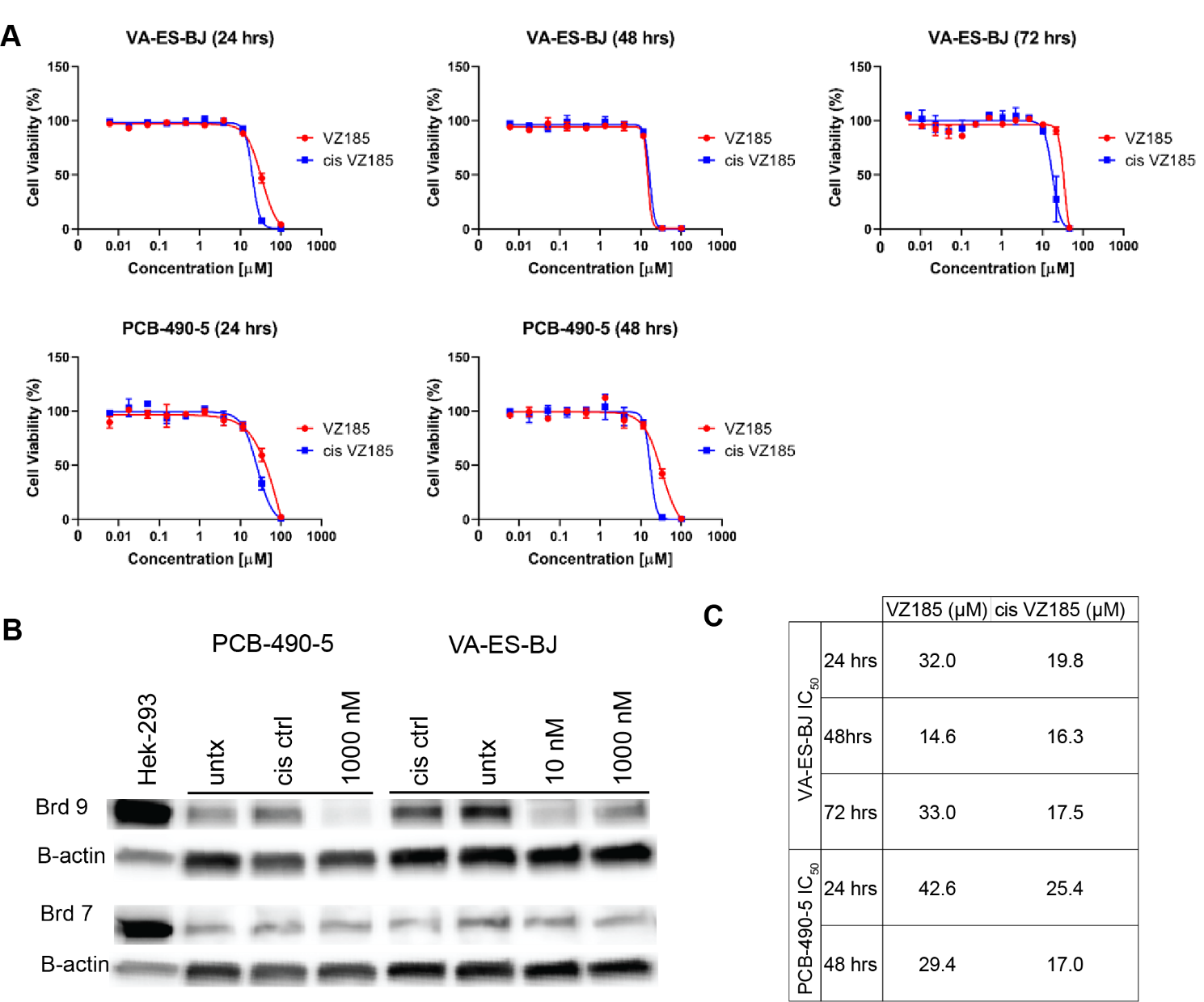
**

**Supplementary Fig 11. Western blot of BRD 7/9 Inhibition as related to Figure 6C. a** The growth inhibition curves for VZ185. **b** The western blot showing the effect of VZ185 on protein levels vs untreated (untx) vs the control CIS-VZ185. **c** The IC_50_ values for panel **b**.


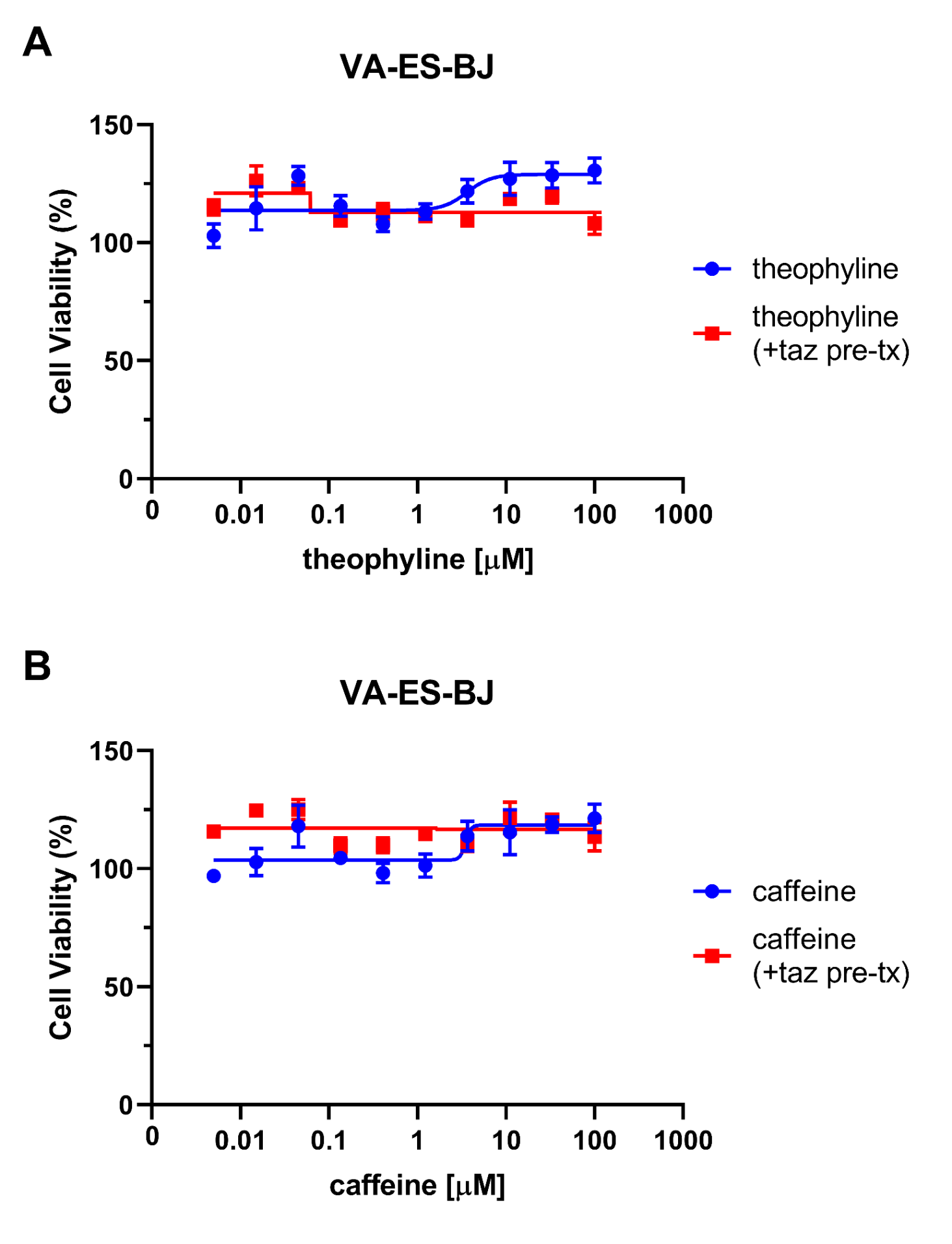
**Supplementary Fig 12.** Targeting the mitochondria in an epithelioid sarcoma cell line VA-ES-BJ, as related to Figure 6A.

**
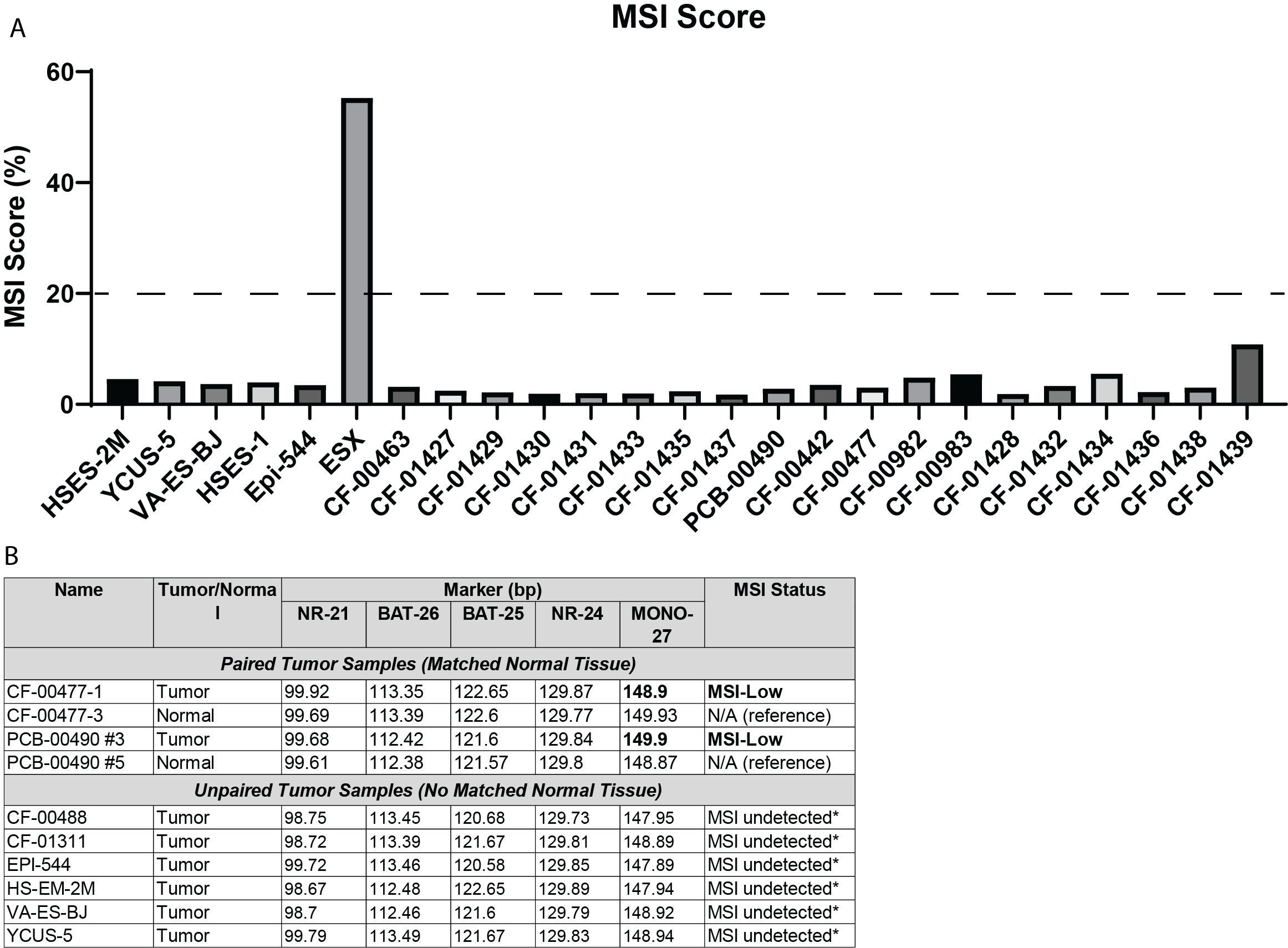
**

**Supplementary Figure 13. Microsatellite Instability Analysis. a** The MSI score as determined by msisensor2 with any results over 20% being MSI high. **b** The MSI results from LabCorp *MSI criteria for unpaired tumor samples: 3 bp shift or more from standard population reference values NR-21 = 98, BAT-26 = 113, BAT-25 = 120, NR-24 = 130, MONO-27 = 150

**
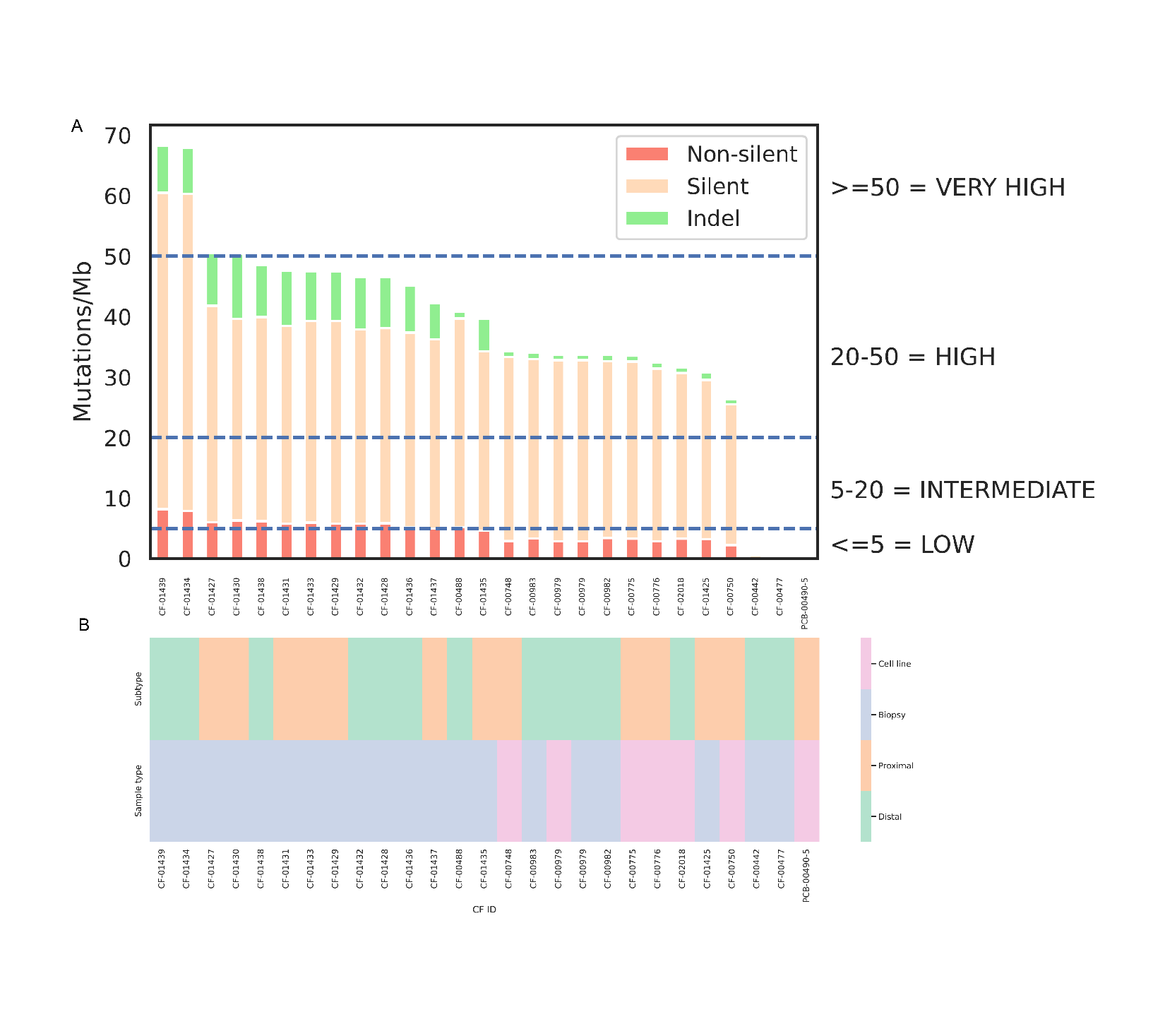
**

**Supplementary Figure 14. Tumor Mutation Burden. a** Tumor Mutation Burden.  **b** Copy Number Variation.

**Supplementary Table 1. Mutation data for *SMARCB1* cell lines**

| Sample Name | Source | Impact | Type | GT | Support | ID | HGVC N | HGVC P |
| --- | --- | --- | --- | --- | --- | --- | --- | --- |
| CF-00748 | - |  | - | - | - | - | - | - |
| CF-00750 | - |  | - | - | - | - | - | - |
| CF-00775 | - |  | - | - | - | - | - | - |
| CF-00776 | - |  | - | - | - | - | - | - |
| CF-00777 | - |  | - | - | - | - | - | - |
| CF-00979 | - |  | - | - | - | - | - | - |
| CF-02018 | Somatic | MODERATE | conservative_inframe_deletion | 0/1 | 613,5 | rs769579890;532969 | c.10_12delATG | p.Met4del |
| PCB-00490-5 | - | - | - | - | - | - | - | - |
| PCB-00495 | Somatic | HIGH | splice_acceptor_variant&intron_variant | 0/1 | 9,4 | rs865871736;COSM1085 | c.629-1G>A | - |

**Supplementary Table 2. Mutation data for *SMARCB1* patient samples**

| **Sample Name** | **Source** | **Impact** | **Type** | **GT** | **Support** | **ID** | **HGVC N** | **HGVC P** |
| --- | --- | --- | --- | --- | --- | --- | --- | --- |
| CF-00442 | Germline | HIGH | bidirectional_gene_fusion (DERL3&SMARCB1) | 0/1 | 22,16 | rs5030614 | n.23834491_23834513delCAACAGGTCATGTTCAATTTCTT | - |
| CF-00477 | Germline | LOW | synonymous_variant | 0/1 | 94,92 | rs2229354;COSM1009;COSM4156041;126368 | c.924G>A | p.Ser308Ser |
| CF-00488 | Somatic | LOW | synonymous_variant | 0/1 | 94,92 | rs2229354;COSM1009;COSM4156041;126368 | c.924G>A | p.Ser308Ser |
| CF-01427 | Somatic | HIGH | stop_gained | 0/1 | 188,9 | - | c.518T>A | p.Leu173* |
| CF-01427 | Somatic | HIGH | frameshift_variant | 0/1 | 185,10 | - | c.508_509insGAGA | p.Asp170fs |
| CF-01427 | Somatic | HIGH | frameshift_variant | 0/1 | 191,10 | - | c.522_525delTCTC | p.Leu175fs |
| CF-01427 | Somatic | MODERATE | disruptive_inframe_deletion | 0/1 | 200,10 | - | c.510_515delTTCCGC | p.Ser171_Ala172del |
| CF-01427 | Somatic | MODERATE | disruptive_inframe_insertion | 0/1 | 191,11 | - | c.520_521insGCGGAA | p.Val174delinsGlyGlyIle |
| CF-01428 | Somatic | MODERATE | missense_variant | 0/1 | 164,3 | rs767791254;COSM285203 | c.310G>A | p.Asp104Asn |
| CF-01428 | Somatic | MODERATE | disruptive_inframe_insertion | 0/1 | 238,3 | - | c.829_830insTCC | p.Asp277delinsValHis |
| CF-01429 | Somatic | MODERATE | missense_variant | 0/1 | 17,2 | - | c.219A>C | p.Lys73Asn |
| CF-01429 | Somatic | HIGH | frameshift_variant | 0/1 | 17,3 | - | c.222_223insCGTG | p.Asn75fs |
| CF-01429 | Somatic | HIGH | frameshift_variant | 0/1 | 17,2 | - | c.224_225insGACG | p.Asn75fs |
| CF-01429 | Somatic | MODERATE | disruptive_inframe_insertion | 0/1 | 18,2 | - | c.227_228insCCT | p.Thr76_Lys77insLeu |
| CF-01429 | Somatic | MODERATE | disruptive_inframe_deletion&splice_region_variant | 0/1 | 17,2 | - | c.230_232delAGG | p.Lys77_Asp78delinsAsn |
| CF-01430 | Somatic | MODERATE | missense_variant | 0/1 | 70,3 | rs768379745 | c.673G>A | p.Asp225Asn |
| CF-01431 | Somatic | MODERATE | conservative_inframe_deletion | 0/1 | 163,6 | rs769579890;532969 | c.10_12delATG | p.Met4del |
| CF-01431 | Somatic | HIGH | frameshift_variant | 0/1 | 312,4 | - | c.508_509insGAGA | p.Asp170fs |
| CF-01431 | Somatic | MODERATE | disruptive_inframe_deletion | 0/1 | 163,6 | - | c.510_515delTTCCGC | p.Ser171_Ala172del |
| CF-01431 | Somatic | HIGH | stop_gained | 0/1 | 312,4 | - | c.518T>A | p.Leu173* |
| CF-01431 | Somatic | MODERATE | disruptive_inframe_insertion | 0/1 | 157,14 | - | c.520_521insGCGGAA | p.Val174delinsGlyGlyIle |
| CF-01431 | Somatic | HIGH | frameshift_variant&splice_region_variant | 0/1 | 312,4 | - | c.522_525delTCTC | p.Leu175fs |
| CF-01432 | Somatic | MODERATE | missense_variant | 0/1 | 56,8 | - | c.219A>C | p.Lys73Asn |
| CF-01432 | Somatic | HIGH | frameshift_variant | 0/1 | 58,9 | - | c.222_223insCGTG | p.Asn75fs |
| CF-01432 | Somatic | HIGH | frameshift_variant | 0/1 | 57,9 | - | c.224_225insGACG | p.Asn75fs |
| CF-01432 | Somatic | MODERATE | disruptive_inframe_insertion | 0/1 | 60,9 | - | c.227_228insCCT | p.Thr76_Lys77insLeu |
| CF-01432 | Somatic | MODERATE | disruptive_inframe_deletion&splice_region_variant | 0/1 | 60,10 | - | c.230_232delAGG | p.Lys77_Asp78delinsAsn |
| CF-01432 | Somatic | MODERATE | missense_variant | 0/1 | 113,5 | - | c.328G>C | p.Val110Leu |
| CF-01432 | Somatic | HIGH | stop_gained&disruptive_inframe_insertion | 0/1 | 112,5 | - | c.331_332insGCTGATGGACACAGCCTTGTA | p.Ser111delinsCysTerTrpThrGlnProCysThr |
| CF-01432 | Somatic | MODERATE | missense_variant | 0/1 | 69,3 | - | c.410A>C | p.Asn137Thr |
| CF-01432 | Somatic | HIGH | frameshift_variant | 0/1 | 74,5 | - | c.413_414insGAATCAACTAATG | p.Ser138fs |
| CF-01432 | Somatic | HIGH | frameshift_variant | 0/1 | 81,2 | - | c.418delC | p.His140fs |
| CF-01432 | Somatic | HIGH | frameshift_variant | 0/1 | 87,2 | - | c.418_425delCACCACTT | p.His140fs |
| CF-01432 | Somatic | HIGH | frameshift_variant | 0/1 | 77,2 | - | c.419_420insGGGATGC | p.His140fs |
| CF-01432 | Somatic | HIGH | frameshift_variant | 0/1 | 79,2 | - | c.424_425insG | p.Leu142fs |
| CF-01432 | Somatic | HIGH | frameshift_variant | 0/1/2 | 76,2,2 | - | c.427_430delGATG | p.Asp143fs |
| CF-01433 | Somatic | MODERATE | conservative_inframe_insertion | 0/1 | 40,2 | - | c.441_442insGGT | p.Cys147_Ser148insGly |
| CF-01434 | Somatic | HIGH | frameshift_variant | 0/1 | 60,3 | - | c.445_457delACAACCATCAACA | p.Thr149fs |
| CF-01434 | Somatic | MODERATE | disruptive_inframe_deletion | 0/1 | 54,3 | - | c.461_463delACC | p.Asn154_Arg155delinsSer |
| CF-01434 | Somatic | MODERATE | conservative_inframe_deletion | 0/1 | 9,34 | - | c.889_906delCTGTGCTCGGAGCTGGGG | p.Leu297_Gly302del |
| CF-01438 | Somatic | HIGH | frameshift_variant | 0/1/2 | 119,2 | - | c.306_307delCA | p.Asn103fs |
| CF-01439 | Somatic | HIGH | frameshift_variant | 0/1 | 118,2 | - | c.323_324delAG | p.Lys108fs |
| CF-01439 | Somatic | HIGH | frameshift_variant | 0/1 | 117,2 | - | c.326_327insCT | p.Val110fs |
| CF-01439 | Somatic | HIGH | frameshift_variant | 0/1 | 117,2 | - | c.329_330insACTTCTCATC | p.Ser111fs |
| CF-01439 | Somatic | HIGH | frameshift_variant | 0/1 | 114,2 | - | c.331_332insTG | p.Ser111fs |
| CF-01439 | Somatic | HIGH | frameshift_variant | 0/1 | 107,3 | - | c.437_438insTGTTG | p.Cys147fs |
| CF-01439 | Somatic | MODERATE | missense_variant | 0/1 | 115,2,3 | - | c.441C>G | p.Cys147Trp |
| CF-01439 | Somatic | MODERATE | disruptive_inframe_deletion | 0/1 | 144,2 | - | c.447_449delAAC | p.Thr150del |
| CF-01439 | Somatic | MODERATE | missense_variant | 0/1 | 125,3 | - | c.449C>G | p.Thr150Ser |
| CF-01439 | Somatic | HIGH | frameshift_variant | 0/1 | 134,3 | - | c.453_457delCAACA | p.Ile151fs |
| CF-01439 | Somatic | MODERATE | missense_variant | 0/1 | 139,3 | - | c.460A>C | p.Asn154His |
| CF-01439 | Somatic | MODERATE | missense_variant | 0/1 | 135,5 | - | c.463C>G | p.Arg155Gly |
| CF-01439 | Somatic | HIGH | frameshift_variant | 0/1 | 139,15 | - | c.508_509insGAGA | p.Asp170fs |
| CF-01439 | Somatic | MODERATE | disruptive_inframe_deletion | 0/1 | 160,14 | - | c.510_515delTTCCGC | p.Ser171_Ala172del |
| CF-01439 | Somatic | HIGH | stop_gained | 0/1 | 144,14 | - | c.518T>A | p.Leu173* |
| CF-01439 | Somatic | MODERATE | disruptive_inframe_insertion | 0/1 | 149,17 | - | c.520_521insGCGGAA | p.Val174delinsGlyGlyIle |
| CF-01439 | Somatic | HIGH | frameshift_variant&splice_region_variant | 0/1 | 151,15 | - | c.522_525delTCTC | p.Leu175fs |
| CF-01439 | Somatic | MODERATE | missense_variant | 0/1 | 300,3 | - | c.707C>T | p.Ala236Val |
| CF-01439 | Somatic | MODERATE | missense_variant | 0/1 | 37,9 | - | c.219A>C | p.Lys73Asn |
| CF-01439 | Somatic | HIGH | frameshift_variant | 0/1 | 40,10 | - | c.222_223insCGTG | p.Asn75fs |
| CF-01439 | Somatic | HIGH | frameshift_variant | 0/1 | 37,9 | - | c.224_225insGACG | p.Asn75fs |
| CF-01439 | Somatic | MODERATE | disruptive_inframe_insertion | 0/1 | 37,9 | - | c.227_228insCCT | p.Thr76_Lys77insLeu |
| CF-01439 | Somatic | MODERATE | disruptive_inframe_deletion&splice_region_variant | 0/1 | 38,10 | - | c.230_232delAGG | p.Lys77_Asp78delinsAsn |
| CF-01439 | Somatic | HIGH | frameshift_variant | 0/1 | 133,8 | - | c.508_509insGAGA | p.Asp170fs |
| CF-01439 | Somatic | MODERATE | disruptive_inframe_deletion | 0/1 | 143,8 | - | c.510_515delTTCCGC | p.Ser171_Ala172del |
| CF-01439 | Somatic | HIGH | stop_gained | 0/1 | 134,9 | - | c.518T>A | p.Leu173* |
| CF-01439 | Somatic | MODERATE | disruptive_inframe_insertion | 0/1 | 133,8 | - | c.520_521insGCGGAA | p.Val174delinsGlyGlyIle |
| CF-01439 | Somatic | HIGH | frameshift_variant&splice_region_variant | 0/1 | 135,8 | - | c.522_525delTCTC | p.Leu175fs |
| CF-01439 | Somatic | HIGH | stop_gained | 0/1 | 174,4 | COSM1226780;COSM996 | c.727C>T | p.Gln243* |

**Supplementary Table 3. Gene ontology classes significantly involved in proximal versus distal epithelioid sarcoma.**

| GO ID | Ontology | qValue | Ratio |
| --- | --- | --- | --- |
| GO:0001525 | angiogenesis | 6.98E-07 | 12/298 |
| GO:0097485 | neuron projection guidance | 8.18E-05 | 9/213 |
| GO:1904018 | positive regulation of vasculature development | 1.73E-04 | 7/115 |
| GO:0043542 | endothelial cell migration | 1.73E-04 | 8/173 |
| GO:0090130 | tissue migration | 2.23E-04 | 9/266 |
| GO:0002040 | sprouting angiogenesis | 4.44E-04 | 6/85 |
| GO:0007409 | axonogenesis | 4.44E-04 | 9/300 |
| GO:0010594 | regulation of endothelial cell migration | 4.69E-04 | 7/147 |
| GO:1901342 | regulation of vasculature development | 4.96E-04 | 8/225 |
| GO:0043534 | blood vessel endothelial cell migration | 5.64E-04 | 6/95 |
| GO:0003158 | endothelium development | 8.29E-04 | 6/103 |
| GO:0010631 | epithelial cell migration | 9.62E-04 | 8/255 |
| GO:0090132 | epithelium migration | 0.001 | 8/261 |
| GO:0031589 | cell-substrate adhesion | 0.0013 | 8/271 |
| GO:0007160 | cell-matrix adhesion | 0.0016 | 6/121 |
| GO:0045765 | regulation of angiogenesis | 0.0019 | 7/203 |
| GO:0010632 | regulation of epithelial cell migration | 0.0019 | 7/203 |
| GO:0090287 | regulation of cell response to GF stimulus | 0.0033 | 7/222 |
| GO:2000146 | negative regulation of cell motility | 0.0033 | 7/223 |
| GO:0045446 | endothelial cell differentiation | 0.0033 | 5/81 |
| GO:0019199 | transmembrane receptor protein kinase activity | 0.003555458 | 5/83 |
| GO:0051271 | negative regulation of cellular component movement | 0.003857359 | 7/234 |
| GO:0001952 | regulation of cell-matrix adhesion | 0.005392731 | 5/92 |
| GO:0019838 | growth factor binding | 0.006116303 | 5/96 |
| GO:0040013 | negative regulation of locomotion | 0.006116303 | 7/256 |
| GO:0021955 | central nervous system neuron axonogenesis | 0.007048111 | 3/15 |
| GO:0019955 | cytokine binding | 0.007048111 | 5/100 |
| GO:0004713 | protein tyrosine kinase activity | 0.007342666 | 5/102 |
| GO:0032102 | negative regulation of response to external stimulus | 0.007821498 | 7/272 |
| GO:0050921 | positive regulation of chemotaxis | 0.008266638 | 5/106 |
| GO:0097529 | myeloid leukocyte migration | 0.008300698 | 6/183 |
| GO:0005003 | ephrin receptor activity | 0.008851803 | 3/17 |
| GO:0001822 | kidney development | 0.010246236 | 5/115 |
| GO:0002064 | epithelial cell development | 0.010246236 | 5/114 |
| GO:0071559 | response to transforming growth factor beta | 0.010246236 | 6/195 |
| GO:0070098 | chemokine-mediated signaling pathway | 0.010246236 | 3/18 |
| GO:0061448 | connective tissue development | 0.010822094 | 5/117 |
| GO:0045785 | positive regulation of cell adhesion | 0.010992216 | 7/299 |
| GO:0043491 | protein kinase B signaling | 0.011223156 | 6/201 |
| GO:1990869 | cellular response to chemokine | 0.016133049 | 4/64 |
| GO:0060326 | cell chemotaxis | 0.017727371 | 6/220 |
| GO:0030336 | negative regulation of cell migration | 0.018977167 | 5/135 |
| GO:1990868 | response to chemokine | 0.022099817 | 4/71 |
| GO:1903035 | negative regulation of response to wounding | 0.022099817 | 4/71 |
| GO:0072001 | renal system development | 0.028914483 | 5/151 |
| GO:0071634 | regulation of transforming growth factor beta production | 0.028914483 | 3/28 |
| GO:0043535 | regulation of blood vessel endothelial cell migration | 0.028914483 | 4/77 |
| GO:0007507 | heart development | 0.031485487 | 6/251 |
| GO:0010810 | regulation of cell-substrate adhesion | 0.032303298 | 5/156 |
| GO:0071604 | transforming growth factor beta production | 0.032303298 | 3/30 |
| GO:1902229 | regulation of intrinsic apoptotic signaling pathway in response to DNA damage | 0.032303298 | 3/30 |
| GO:0001655 | urogenital system development | 0.032386543 | 5/158 |
| GO:0060562 | epithelial tube morphogenesis | 0.03371994 | 5/160 |
| GO:0030099 | myeloid cell differentiation | 0.036944635 | 6/264 |
| GO:0002685 | regulation of leukocyte migration | 0.037129725 | 5/165 |
| GO:0050673 | epithelial cell proliferation | 0.037129725 | 6/266 |
| GO:0052548 | regulation of endopeptidase activity | 0.039583671 | 6/270 |
| GO:0071560 | cellular response to transforming growth factor beta stimulus | 0.039868234 | 5/169 |
| GO:0030335 | positive regulation of cell migration | 0.040750991 | 6/274 |
| GO:0035850 | epithelial cell differentiation involved in kidney development | 0.040750991 | 3/34 |
| GO:0021954 | central nervous system neuron development | 0.042406767 | 3/35 |
| GO:0002686 | negative regulation of leukocyte migration | 0.042406767 | 3/35 |
| GO:0050900 | leukocyte migration | 0.044531784 | 6/281 |
| GO:0050920 | regulation of chemotaxis | 0.044875838 | 5/177 |
| GO:0061005 | cell differentiation involved in kidney development | 0.051822356 | 3/38 |
| GO:0072132 | mesenchyme morphogenesis | 0.055179077 | 3/39 |
| GO:0052547 | regulation of peptidase activity | 0.058631933 | 6/299 |
| GO:0030595 | leukocyte chemotaxis | 0.058768516 | 5/190 |
| GO:0051896 | regulation of protein kinase B signaling | 0.0593459 | 5/191 |
| GO:0002688 | regulation of leukocyte chemotaxis | 0.062289734 | 4/105 |
| GO:0032835 | glomerulus development | 0.062289734 | 3/42 |
| GO:0071622 | regulation of granulocyte chemotaxis | 0.062289734 | 3/42 |
| GO:0071621 | granulocyte chemotaxis | 0.062289734 | 4/105 |
| GO:0045861 | negative regulation of proteolysis | 0.06237922 | 5/196 |
| GO:1902743 | regulation of lamellipodium organization | 0.065044807 | 3/43 |
| GO:0010634 | positive regulation of epithelial cell migration | 0.065280848 | 4/108 |
| GO:0090092 | regulation of transmembrane receptor protein kinase signaling pathway | 0.06613659 | 5/201 |
| GO:0050819 | negative regulation of coagulation | 0.06613659 | 3/44 |
| GO:1900047 | negative regulation of hemostasis | 0.06613659 | 3/44 |
| GO:0004714 | transmembrane receptor protein tyrosine kinase activity | 0.069834236 | 3/45 |
| GO:0071675 | regulation of mononuclear cell migration | 0.073635812 | 3/46 |
| GO:0031345 | negative regulation of cell projection organization | 0.082131571 | 4/117 |
| GO:0072109 | glomerular mesangium development | 0.082532181 | 2/10 |
| GO:0005126 | cytokine receptor binding | 0.082532181 | 5/214 |
| GO:0060485 | mesenchyme development | 0.084966124 | 5/216 |
| GO:1903034 | regulation of response to wounding | 0.086225591 | 4/120 |
| GO:0008630 | intrinsic apoptotic signaling pathway in response to DNA damage | 0.087810288 | 3/50 |
| GO:0030194 | positive regulation of blood coagulation | 0.090308971 | 2/11 |
| GO:0030193 | regulation of blood coagulation | 0.090308971 | 3/51 |
| GO:0061045 | negative regulation of wound healing | 0.090308971 | 3/52 |
| GO:0097530 | granulocyte migration | 0.090308971 | 4/123 |
| GO:2001212 | regulation of vasculogenesis | 0.090308971 | 2/11 |
| GO:1903427 | negative regulation of reactive oxygen species biosynthetic process | 0.090308971 | 2/11 |
| GO:2000377 | regulation of reactive oxygen species metabolic process | 0.090308971 | 4/123 |
| GO:0010633 | negative regulation of epithelial cell migration | 0.090308971 | 3/52 |
| GO:0010595 | positive regulation of endothelial cell migration | 0.099900279 | 3/54 |

**Supplementary Table 4. STR profiles**

|  |  | Cell line | | | | | | |
| --- | --- | --- | --- | --- | --- | --- | --- | --- |
|  |  | PCB-490-5 | VA-ES-BJ | YCUS-5 | CF-01311 | EPI-544 | HS-ES-1 | HS-ES-2R |
| STR Feature | D3S1358 | 14 | 16 | 15 | 17 | 15 | 17 | 15 |
|  |  | 17 | 16 | 16 |  | 16 | 18 | 15 |
|  | TH01 | 8 | 6 | 6 | 7 | 7 | 7 | 7 |
|  |  | 8 | 6 | 9 | 9.3 | 9 | 9 | 9 |
|  | D21S11 | 29 | 27 | 29 | 28 | 30 | 29 | 30 |
|  |  | 31 | 28 | 30 | 31.2 | 33.2 | 30 | 31 |
|  | D18S51 | 13 | 12 | 17 | 18 | 13 | 13 | 13 |
|  |  | 15 | 13 | 20 |  | 15 | 14 | 17 |
|  | Penta_E | 13 | 13 | 11 | 15 | 14 | 17 | 15 |
|  |  | 15 | 13 | 16 | 19 |  | 20 | 20 |
|  | D5S818 | 11 | 11 | 9 | 10 | 10 | 10 | 11 |
|  |  | 12 | 13 | 11 | 11 | 13 | 10 | 11 |
|  | D13S317 | 12 | 11 | 11 | 11 | 8 | 9 | 12 |
|  |  | 14 | 12 | 11 |  | 10 | 9 | 14 |
|  | D7S820 | 8 | 11 | 11 | 8 | 10 | 8 | 8 |
|  |  | 10 | 12 | 11 | 10 | 13 | 12 | 8 |
|  | D16S539 | 13 | 11 | 9 | 9 | 13 | 10 | 10 |
|  |  | 13 | 12 | 9 | 12 | 13 | 10 | 11 |
|  | CSF1PO | 11 | 11 | 10 | 11 | 10 | 11 | 10 |
|  |  | 11 | 12 | 11 | 12 | 11 | 13 | 12 |
|  | Penta_D | 10 | 10 | 9 | 9 |  | 9 | 9 |
|  |  | 10 | 13 | 11 |  |  | 12 | 10 |
|  | AMEL | X | X | X | X | X | X | X |
|  |  | X | X | X |  | Y | Y | Y |
|  | vWA | 15 | 18 | 14 | 16 | 14 | 18 | 18 |
|  |  | 16 | 19 | 16 |  | 14 | 18 | 18 |
|  | D8S1179 | 11 | 11 | 11 | 13 | 15 | 11 | 10 |
|  |  | 14 | 15 | 15 | 15 | 16 | 16 | 15 |
|  | TPOX | 9 | 8 | 8 | 8 | 8 | 8 | 8 |
|  |  | 11 | 8 | 8 | 11 | 8 | 8 | 8 |
|  | FGA | 20 | 22 | 23 | 22 | 23 | 22 | 23 |
|  |  | 24 | 22 | 23 | 23 | 24 | 22 | 23 |
